# LIMK2-NKX3.1 Engagement Promotes Castration-Resistant Prostate Cancer

**DOI:** 10.3390/cancers13102324

**Published:** 2021-05-12

**Authors:** Moloud A. Sooreshjani, Kumar Nikhil, Mohini Kamra, Dung N. Nguyen, Dinesh Kumar, Kavita Shah

**Affiliations:** Department of Chemistry and Purdue University Center for Cancer Research, Purdue University, 560 Oval Drive, West Lafayette, IN 47907, USA; maflakis@purdue.edu (M.A.S.); niksbiotech@gmail.com (K.N.); mkamra@purdue.edu (M.K.); nguye445@purdue.edu (D.N.N.); kumar516@purdue.edu (D.K.)

**Keywords:** LIMK2, castration-resistant prostate cancer, NKX3.1

## Abstract

**Simple Summary:**

Prostate cancer is the principal cause of cancer-related mortality in men. While localized tumors can be successfully treated by orchiectomy or medical castration, most of the patients ultimately progress to the castration-resistant prostate cancer (CRPC) stage, which is incurable at present. Thus, uncovering the underlying mechanisms that cause CRPC could result in promising therapeutics. Our laboratory has identified LIMK2 kinase as an actionable target for CRPC. LIMK2 is vastly expressed in CRPC but minimally in normal prostates. LIMK2 knockout mice are healthy, indicating that LIMK2 inhibition should have minimal toxicity. LIMK2 is also expressed in other aggressive cancers; however, the molecular mechanisms leading to malignancy remain mostly unknown. This study identified that LIMK2 downregulates a prostate-specific tumor suppressor protein-NKX3.1 using two mechanisms. NKX3.1 loss is strongly associated with prostate cancer. Thus, LIMK2 inhibitor provides a powerful opportunity to rescue NKX3.1 loss, thereby preventing and/or delaying prostate cancer progression.

**Abstract:**

NKX3.1’s downregulation is strongly associated with prostate cancer (PCa) initiation, progression, and CRPC development. Nevertheless, a clear disagreement exists between NKX3.1 protein and mRNA levels in PCa tissues, indicating that its regulation at a post-translational level plays a vital role. This study identified a strong negative relationship between NKX3.1 and LIMK2, which is critical in CRPC pathogenesis. We identified that NKX3.1 degradation by direct phosphorylation by LIMK2 is crucial for promoting oncogenicity in CRPC cells and in vivo. LIMK2 also downregulates NKX3.1 mRNA levels. In return, NKX3.1 promotes LIMK2’s ubiquitylation. Thus, the negative crosstalk between LIMK2-NKX3.1 regulates AR, ARv7, and AKT signaling, promoting aggressive phenotypes. We also provide a new link between NKX3.1 and PTEN, both of which are downregulated by LIMK2. PTEN loss is strongly linked with NKX3.1 downregulation. As NKX3.1 is a prostate-specific tumor suppressor, preserving its levels by LIMK2 inhibition provides a tremendous opportunity for developing targeted therapy in CRPC. Further, as NKX3.1 downregulates AR transcription and inhibits AKT signaling, restoring its levels by inhibiting LIMK2 is expected to be especially beneficial by co-targeting two driver pathways in tandem, a highly desirable requisite for developing effective PCa therapeutics.

## 1. Introduction

Prostate cancer (PCa) is among the most prevalent noncutaneous cancers and one of the leading causes of cancer-related death in men in the western world [1,2]. Androgen deprivation therapy (ADT), which inhibits the androgen receptor (AR) signaling, is considered the gold standard treatment for PCa. However, ADT has a temporary effect and most of the patients ultimately develop castration-resistant prostate cancer (CRPC) [3]. CRPC is a heterogeneous cancer that has a poor prognosis, with 9–36 months of median survival. Despite advanced treatment options, such as the development of second-generation AR antagonists, the majority of patients develop drug resistance. Therefore, uncovering the underlying mechanisms that drive CRPC is an important step for developing new therapies and improving patient life expectancy.

We recently identified LIM kinase2 (LIMK2) as an actionable target in CPRC [4]. LIMK2 levels increase in mouse prostates following orchiectomy. Likewise, normal human prostate specimens showed negligible LIMK2 levels but were very high in CRPC tissues [4]. Most importantly, we showed that conditional knockdown of LIMK2 in vivo completely reverses tumorigenesis, emphasizing its potential as a drug target in CRPC. LIMK2 is also upregulated in multiple other cancers and promotes tumorigenesis and metastasis [5,6,7,8]. Despite these reports, the downstream mechanisms by which LIMK2 induces malignancy are largely unknown as only a few of its substrates are known to date. Recently, we identified TWIST1, SPOP, and PTEN as direct LIMK2 substrates, which shed light on the molecular mechanisms by which LIMK2 triggers CRPC pathogenesis [4,9,10].

This study focuses on the tumor-suppressor NKX3.1, which was uncovered as a direct substrate of LIMK2 using an inventive screen [11,12,13]. NKX3.1 is a prostate-specific homeodomain-containing transcription factor, which is expressed specifically in prostate luminal epithelia. Androgen receptor (AR) signaling maintains NKX3.1 expression in postnatal and adult prostates [14]. NKX3.1 promotes cell differentiation, maintenance, and lineage plasticity, and its genetic loss inhibits ductal branching and production of secretory proteins. Loss of NKX3.1 is a key initiating event in human PCa development [15]. Homozygous deletion of nkx3.1 in mice results in prostate epithelial hyperplasia, which rapidly progresses to PIN-like lesions in a year or two [16]. Germ-line mutations in NKX3.1 affect its DNA-binding ability and are associated with an increased risk of PCa [17]. Similarly, NKX3.1 is either reduced or lacking in about 50% of PIN and primary PCa human tumors, which increases to more than 80% in metastatic cases [18]. Consistent with its tumor-suppressor functions, overexpression of NKX3.1 inhibits cell proliferation and mbvcxcolony formation in cells and tumorigenesis in vivo [19]. In this study, we uncovered a key bidirectional relationship between LIMK2 and NKX3.1, which plays a critical role in CRPC progression.

## 2. Results

### 2.1. Analog-Sensitive LIMK2 (LIMK2-as7) Identifies NKX3.1 as a Direct Target

**Figure 1:**
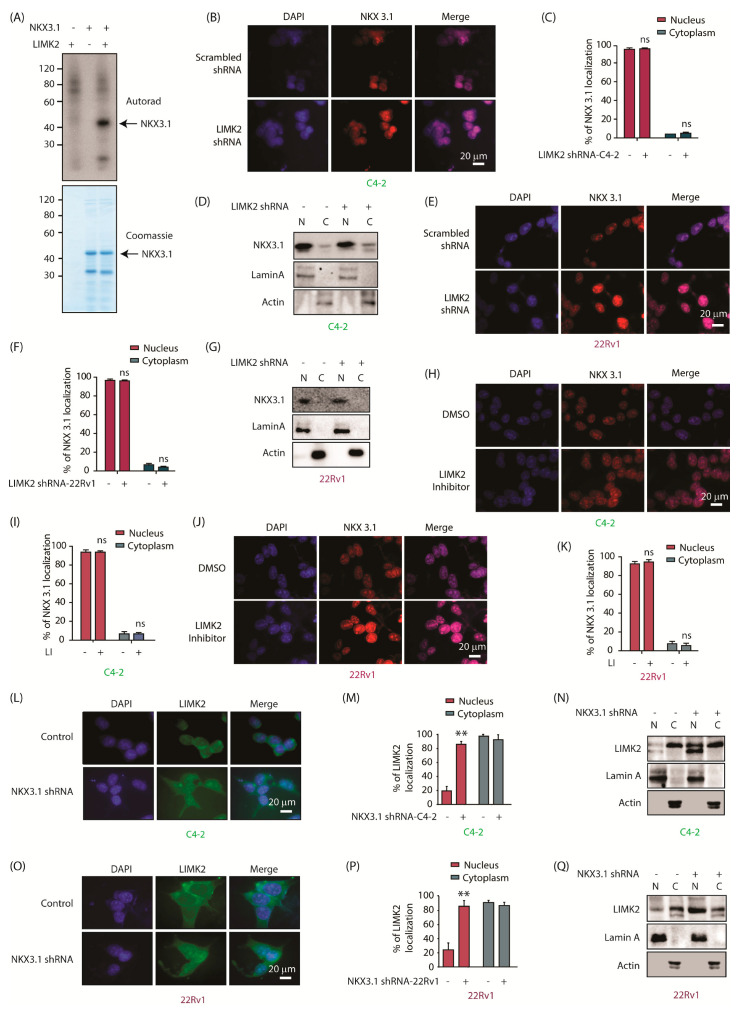
LIMK2 directly phosphorylates NKX3.1 and in turn, NKX3.1 regulates LIMK2 localization. (**A**) LIMK2 and NKX3.1 were incubated with [γ-^32^P] ATP. The mixtures were separated by SDS-PAGE. NKX3.1 phosphorylation was detected by autoradiography. The gel was stained with Coomassie Brilliant Blue. The upper panel shows an autoradiograph. The lower panel shows a gel stained with Coomassie blue. In lane 1, LIMK2 was incubated with radiolabeled ATP and without NKX3.1. In lane 2, NKX3.1 was incubated with radiolabeled ATP and without LIMK2. In lane 3, NKX3.1 was incubated with radiolabeled ATP and LIMK2. The in vitro kinase assay was repeated three times; (**B**) LIMK2 depletion does not impact the subcellular localization of NKX3.1 in C4-2 cells. Immunofluorescence micrographs of C4-2 cells infected with scrambled and LIMK2 shRNA were probed with NKX3.1 antibody (red). Nuclear counterstain is represented by DAPI (blue). [Scale bar = 20 μm]; (**C**) Quantification of subcellular localization based on analysis of micrographs in B. In total, 100 cells were counted from 20 different frames; (**D**) Treatment of C4-2 cells with LIMK2 shRNA lentivirus did not change NKX3.1 localization. C4-2 cells were treated with LIMK2 shRNA lentivirus, and NKX3.1 location was analyzed using fractionation. Actin and lamin A were used as cytoplasmic and nuclear controls, respectively. The experiment was performed three times; (**E**) LIMK2 depletion does not impact the subcellular localization of NKX3.1 in 22Rv1 cells. Immunofluorescence analysis of subcellular localization of NKX3.1 (red) in 22Rv1 cells with and without LIMK2 knockdown. DAPI (blue) is used for nuclear counterstain. [Scale bar = 20 μm]; (**F**) Quantification of data obtained from E. A total of 100 cells were counted from the analysis of 20 different frames; (**G**) Treatment of 22Rv1 cells with LIMK2 shRNA lentivirus did not change NKX3.1 localization. 22Rv1 cells were treated with LIMK2 shRNA lentivirus, and NKX3.1 localization was analyzed. The experiment was performed three times; (**H**) LIMK2 inhibition does not affect the subcellular localization of NKX3.1 in C4-2 cells. Immunofluorescence analysis of subcellular distribution of NKX3.1 (red) in response to LIMK2 inhibitor in C4-2 cells. The blue panel represents DAPI as the nuclear counterstain. [Scale bar = 20 μm]; (**I**) Quantification of data obtained in H from 20 different frames (total 100 cells); (**J**) Inhibition of LIMK2 kinase activity does not change the subcellular localization of NKX3.1 in 22Rv1 cells. Images obtained from immunofluorescence microscopy of 22Rv1 cells. Red-NKX3.1, blue-DAPI. [Scale bar = 20 μm]; (**K**) Quantification of subcellular distribution of NKX3.1 in 22Rv1 cells from 20 different frames (total 100 cells) as shown in J; (**L**) Treatment of C4-2 cells with NKX3.1 shRNA lentivirus caused the distribution of LIMK2 from the cytosol to the nucleus. LIMK2 (green) and nucleus (blue). C4-2 cells were stained with an anti-NKX3.1 monoclonal antibody, followed by an FITC-conjugated antibody. Nuclei were stained with DAPI. Scale bar equals 20 µM. (**M**) Quantification of LIMK2 localization in response to the NKX3.1 shRNA lentivirus in C4-2 cells is shown in L. 100 cells were counted from several random frames. Bar graph shows the mean number of counted cells. The data is shown as the number of cells ± SD. The experiment was performed three times. ** *p* < 0.01; (**N**) Subcellular fractionation of LIMK2 in C4-2 cells in response to knockdown of NKX3.1. C4-2 cells were treated with scrambled or NKX3.1 shRNA lentivirus; (**O**) Subcellular localization of LIMK2 in 22Rv1 cells infected by NKX3.1 shRNA was visualized using immunofluorescence. LIMK2 (green) and nucleus (blue) [Scale bar = 20 μm]; (**P**) Quantification of LIMK2 localization in response to the NKX3.1 shRNA lentivirus in 22Rv1 cells as presented in (**O**). 100 cells were counted from many random frames. The experiment was performed three times. ** *p* < 0.01; (**Q**) Subcellular fractionation of LIMK2 in 22Rv1 cells in response to the NKX3.1 knockdown.

Previously, we developed a chemical methodology to identify the direct substrates of any kinase of interest. This method utilizes a functionally silent space-creating mutation in the active site of the kinase, which makes it susceptible to an ATP analog bearing the complementary bulky substituents, such as benzyl or phenethyl at the N-6 position. The pocket in the kinase is created by mutating the conserved gatekeeper residue to a glycine residue (analog-sensitive1 aka-as1, example CDK5-as1). While most of the kinases only require a mandatory gatekeeper mutation, many other kinases require additional mutations [20]. We identified one such residue L194 in the subdomain IV of Aurora kinase A (AURKA) active site, which needed to be mutated to valine (L194V) in addition to the gatekeeper mutation. This second mutation rendered both Aurora A (L194V, L210G) and Aurora B (L138V, L154G) kinases highly receptive to N-6-modified ATP analogs, which were named as analog-sensitive-7 aka as7 kinases (AURKA-as7 and AURKB-as7). In contrast, the corresponding AURKA-as1 (L210G) and AURKB-as1 (L154G) showed poor K_cat_ with N-6-Phenethyl ATP [12]. Using Aurora A-as7 kinase, we have identified several direct targets, including PHLDA1, LIMK2, ALDH1A1, SPOP, YBX1, and TWIST1, in cancer cells [5,12,21,22,23,24]. Based on the same engineering criteria, we generated the corresponding LIMK2-as7 kinase (L389V, T405G), which showed high catalytic activity with N-6-Phenethyl-ATP. Using LIMK2-as7, we previously identified SPOP and PTEN as its direct targets [9,10]. In this study, we focused on NKX3.1, which was identified in a global screen. To confirm this observation, we performed an in vitro kinase assay using 6x-His-NKX3.1. LIMK2 robustly phosphorylates NKX3.1 in vitro (Figure 1A).

### 2.2. LIMK2 Does Not Alter NKX3.1 Subcellular Localization, but NKX3.1 Promotes Cytoplasmic Localization of LIMK2

To probe the relationship between LIMK2 and NKX3.1, two CRPC cell lines, 22Rv1 and C4-2, were utilized. NKX3.1 was predominantly nuclear as expected (Figure 1B,C). LIMK2 depletion showed no change in NKX3.1 localization in C4-2 cells (Figure 1B,C). To confirm this result, we also performed nuclear and cytoplasmic fractionation of scrambled shRNA-treated and LIMK2-knocked down cells, which showed minimal change in NKX3.1 localization upon LIMK2 depletion (Figure 1D). We obtained the same results in 22Rv1 cells, confirming that LIMK2 does not regulate NKX3.1 localization in CRPC cells (Figure 1E–G). We further confirmed these results using an LIMK2 inhibitor-N-benzyl-N-ethyl-4-(N-phenylsulfamoyl)benzamide (LI) [25]. The IC_50_ of LI was found to be 2.07 and 2.17 µM in C4-2 and 22Rv1 cells, respectively, when treated for 48 h (Figure S5). Thus, LIMK2 was inhibited for 12 h at the 10 µM concentration and its impact on the subcellular location of NKX3.1 analyzed. Similar to the results obtained upon LIMK2 silencing, LIMK2 inhibition did not change the nuclear localization of NKX3.1 in both C4-2 and 22Rv1 cells (Figure 1H–K). Together, these data show that LIMK2 does not regulate the subcellular residence of NKX3.1.

As observed before, LIMK2 was predominantly cytoplasmic with some nuclear residence in C4-2 cells (4, 9, 10). NKX3.1 depletion caused robust nuclear translocation of LIMK2, which was further confirmed by subcellular fractionation (Figure 1L–N). 22Rv1 cells showed similar nuclear migration upon NKX3.1 silencing, indicating that NKX3.1 promotes cytoplasmic residence of LIMK2 in CRPC cells (Figure 1O–Q). Figure S1 shows raw data for Figure 1D,G,N,Q.

### 2.3. LIMK2 Negatively Regulates NKX3.1

**Figure 2:**
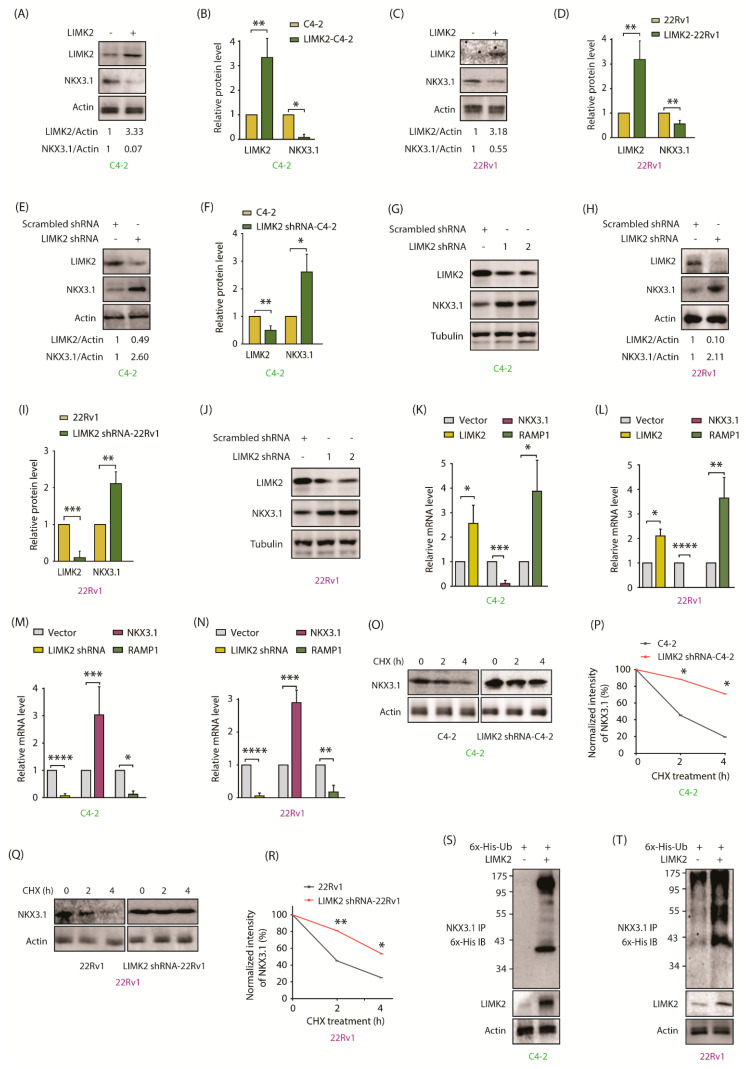
LIMK2 negatively regulates NKX3.1 levels. (**A**) Overexpression of LIMK2 decreases NKX3.1 protein level in C4-2 cells. C4-2 cells were infected with LIMK2 retrovirus and harvested after 33 h; (**B**) Quantitative analysis of LIMK2 and NKX3.1 protein levels from three independent experiments. * *p* < 0.05, ** *p* < 0.01; (**C**) LIMK2 overexpression decreases NKX3.1 protein level in 22Rv1 cells; (**D**) Quantitative analysis of LIMK2 and NKX3.1 protein levels from three independent experiments. ** *p* < 0.01; (**E**) Downregulation of LIMK2 increases NKX3.1 protein level in C4-2 cells. C4-2 cells were infected with LIMK2 shRNA lentivirus. Actin was used as a control; (**F**) Quantitative analysis of LIMK2 and NKX3.1 protein levels from three independent experiments. * *p* < 0.05, ** *p* < 0.01; (**G**) LIMK2 depletion using two different shRNAs increased NKX3.1 levels in C4-2 cells; (**H**) Downregulation of LIMK2 increases NKX3.1 protein level in 22Rv1 cells; (**I**) Quantitative analysis of LIMK2 and NKX3.1 protein levels from three independent experiments using scrambled shRNA and LIMK2-shRNA-infected 22Rv1 cells. ** *p* < 0.01, *** *p* < 0.001; (**J**) LIMK2 depletion using two different shRNAs increased NKX3.1 levels in 22Rv1 cells; (**K**) LIMK2 overexpression reduced the mRNA levels of NKX3.1 in C4-2 and (**L**) 22Rv1 cells. The target cells were treated with LIMK2 retrovirus. Total RNA was isolated and mRNA levels were measured by qRT-PCR. GAPDH was used as the control. The data were from three independent experiments. * *p* < 0.05, ** *p* < 0.01, *** *p* < 0.001, and **** *p* < 0.0001; (**M**) shRNA-mediated knockdown of LIMK2 increased NKX3.1 mRNA level in C4-2 and (**N**) 22Rv1 cells. GAPDH was used as the control. Data were obtained from three independent experiments. * *p* < 0.05, ** *p* < 0.01, *** *p* < 0.001, and **** *p* < 0.0001; (**O**) NKX3.1 degradation in C4-2 cells treated with LIMK2 shRNA and cycloheximide (CHX). We treated C4-2 cells with LIMK2 shRNA lentivirus for 33 h, followed by CHX treatment (20 µg/mL) for 2 and 4 h. Ethanol was used as a vehicle control for CHX; (**P**) Dot plot showing relative degradation of NKX3.1 protein in scrambled shRNA-treated and LIMK2 knockdown-C4-2 cells from three independent experiments. * *p* < 0.05; (**Q**) NKX3.1 degradation in 22Rv1 cells treated with LIMK2 shRNA lentivirus; (**R**) Dot plot showing relative degradation of NKX3.1 protein in scrambled shRNA-treated and LIMK2 knockdown-22Rv1 cells from three independent experiments. * *p* < 0.05, ** *p* < 0.01; (**S**) LIMK2 overexpression causes ubiquitylation of NKX3.1 in C4-2 cells; (**T**) LIMK2 overexpression causes ubiquitylation of NKX3.1 in 22Rv1 cells.

NKX3.1 is downregulated upon castration in mice, which is consistent with its origin as an androgen-responsive gene [26]. We showed that LIMK2 levels increase upon castration due to increased hypoxia [4]. Therefore, we suspected that LIMK2 may also be responsible for NKX3.1 downregulation. To check this hypothesis, we infected C4-2 cells with LIMK2 retrovirus transiently, which caused significant downregulation of NKX3.1 (Figure 2A,B). Consistent with this finding, overexpression of LIMK2 reduced expression of NKX3.1 in 22Rv1 cells as well (Figure 2C,D). Besides, LIMK2 knockdown using the corresponding shRNA in C4-2 cells enhanced NKX3.1 levels (Figure 2E,F). To confirm specificity, we also used additional LIMK2 shRNA to investigate its impact on NKX3.1 levels, which confirmed that LIMK2 negatively regulates NKX3.1 (Figure 2G). Analogous results were obtained in 22Rv1 cells (Figure 2H–J). Thus, these results indicate that LIMK2 negatively impacts NKX3.1.

### 2.4. LIMK2 Negatively Regulates NKX3.1 mRNA Levels and Decreases It’s Stability by Enhanced Ubiquitylation

To uncover the mechanistic basis of NKX3.1 downregulation by LIMK2, we first analyzed the mRNA levels of NKX3.1 level in response to LIMK2 overexpression in C4-2 and 22Rv1 cells by qRT-PCR, which revealed massive downregulation of NKX3.1 mRNAs in both cell lines (Figure 2K,L). This result was confirmed by silencing LIMK2 in C4-2 and 22Rv1 cells, which resulted in about 3-fold increase in NKX3.1 mRNA levels (Figure 2M,N). As NKX3.1 directly represses RAMP1 transcription [27], RAMP1 mRNA levels were used as control, which increased when NKX3.1 mRNA levels decreased and vice versa. These results confirm that LIMK2 downregulates NKX3.1 mRNA levels.

As LIMK2 directly phosphorylates NKX3.1, we hypothesized that LIMK2 may also regulate the protein stability of NKX3.1. C4-2 and LIMK2-depleted C4-2 cells were exposed to cycloheximide to prevent protein synthesis, and the NKX3.1 degradation profile compared, which revealed a robust increase in NKX3.1 stability when LIMK2 was reduced (Figure 2O,P). LIMK2 knockdown in 22Rv1 cells also increased NKX3.1 stability, thereby confirming that LIMK2-mediated NKX3.1 downregulation is a common mechanism in CRPC cells (Figure 2Q,R). We next examined whether LIMK2-mediated NKX3.1 degradation was dependent or independent of ubiquitin. LIMK2 overexpression indeed resulted in enhanced ubiquitylation of NKX3.1 in C4-2 and 22Rv1 cells (Figure 2S,T), thereby confirming that LIMK2 regulates NKX3.1 both at transcriptional and post-translational stages. Figure S2 shows raw data for Figure 2A,C,E,G,H,J,O,Q,S,T.

### 2.5. NKX3.1 Negatively Regulates LIMK2 at mRNA and Protein Levels

**Figure 3:**
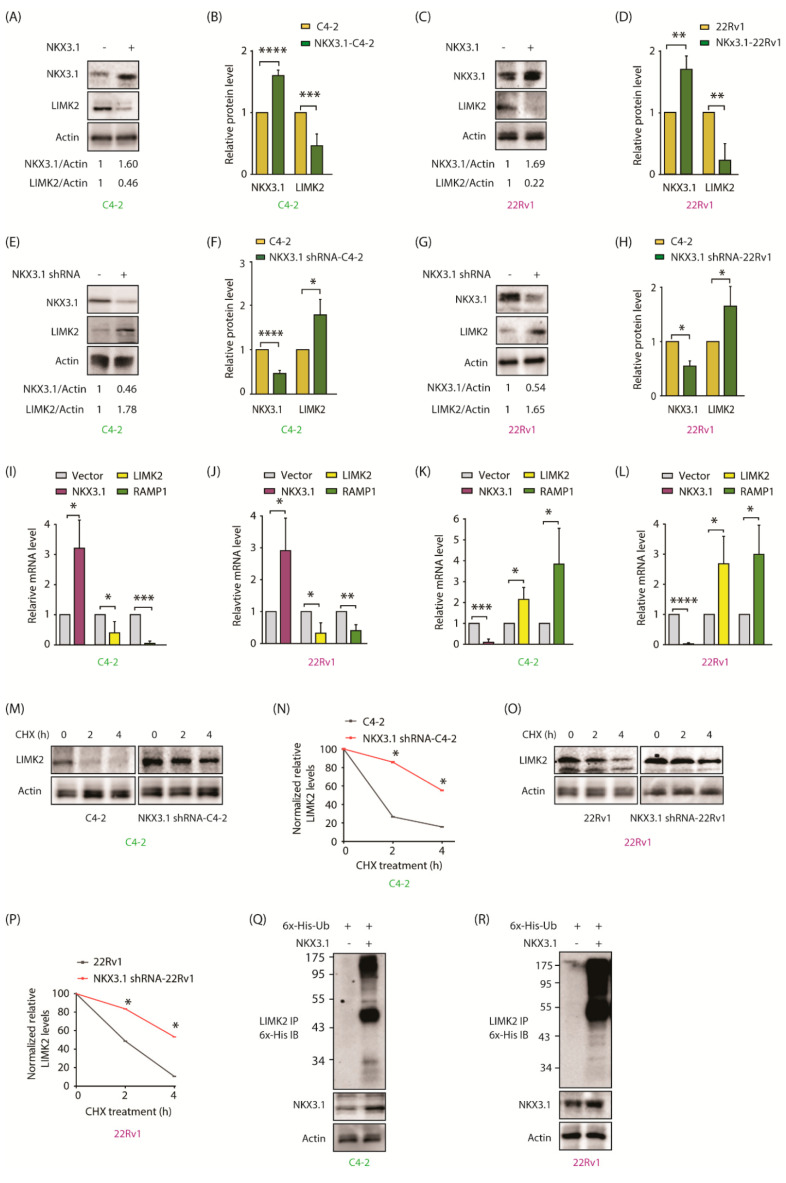
NKX3.1 negatively regulates LIMK2 mRNA levels and protein stability. (**A**) NKX3.1 overexpression results in LIMK2 protein downregulation. C4-2 cells were transiently infected using the either vector or NKX3.1 retrovirus; (**B**) The histogram represents a quantitative analysis of protein levels from three independent experiments. The protein levels are normalized to the actin. *** *p* < 0.001 and **** *p* < 0.0001; (**C**) NKX3.1 upregulation results in LIMK2 protein downregulation. 22Rv1 cells were infected with NKX3.1 retrovirus for 33 h; (**D**) The graph shows quantitative analysis of protein levels from three independent experiments. ** *p* < 0.01; (**E**) Downregulation of NKX3.1 increases LIMK2 protein level. C4-2 cells were transiently infected with NKX3.1 shRNA lentivirus; (**F**) The histogram shows quantitative analysis of LIMK2 and NKX3.1 protein levels in scrambled shRNA-treated and NKX3.1 shRNA-treated cells from three independent experiments. * *p* < 0.05, **** *p* < 0.0001; (**G**) Downregulation of NKX3.1 increases LIMK2 protein level in 22Rv1 cells; (**H**) Quantitative analysis of detected protein levels from three independent experiments. * *p* < 0.05; (**I**) Ectopic expression of NKX3.1 reduces LIMK2 mRNA level in C4-2 and (**J**) 22Rv1 cells. The target cells were infected with NKX3.1 retrovirus. Total RNA was extracted, and mRNA levels were quantified by qRT-PCR from three independent experiments. GAPDH was used as the control. * *p* < 0.05, ** *p* < 0.01, and *** *p* < 0.001; (**K**) Depletion of NKX3.1 increases LIMK2 mRNA level in C4-2 cells and (**L**) 22Rv1 cells. Data were obtained from three independent experiments using GAPDH as the control. * *p* < 0.05, *** *p* < 0.001, and **** *p* < 0.0001; (**M**) LIMK2 degradation in C4-2 cells upon NKX3.1 knockdown. The cells were treated by NKX3.1 shRNA lentivirus for 33 h, followed by CHX (20 µg/mL) treatment for 2 and 4 h. Subsequently, the cells were lysed, and the LIMK2 protein level was analyzed; (**N**) The graph represents the statistical analysis from three independent experiments as described in (**M**). * *p* < 0.05; (**O**) LIMK2 degradation in 22Rv1 cells treated with NKX3.1 shRNA lentivirus and CHX. (**P**) The graph represents the statistical analysis from three independent experiments as treated in (**O**); * *p* < 0.05. (**Q**) NKX3.1 overexpression increases LIMK2 ubiquitylation in C4-2 and (**R**) 22Rv1 cells. Target cells expressing either vector or NKX3.1 were infected by 6x-His-ubiquitin retrovirus. Cells were treated with MG132 for 10 h. Ubiquitylation of LIMK2 was analyzed using 6x-His antibody.

Many substrates regulate their regulator kinases in a feedback loop. Our previous studies have shown that LIMK2 substrates—TWIST1, SPOP, and PTEN—regulate LIMK2 in a feedback loop [4,9,10]. Therefore, we investigated whether such a loop exists between NKX3.1 and LIMK2. We overexpressed NKX3.1 in C4-2 and 22Rv1 cells, which caused robust downregulation of LIMK2 in both cell types (Figure 3A–D). Similarly, when NKX3.1 was knocked-down in CRPC cells, LIMK2 levels increased substantially, confirming the reciprocal cross talk (Figure 3E–H). 

As NKX3.1 is a transcription factor, we examined whether it upregulates LIMK2 at the transcriptional stage. C4-2 and 22Rv1 cell lines were infected with NKX3.1 retrovirus. Overexpression of NKX3.1 induced a robust reduction in LIMK2 mRNA levels in both C4-2 and 22Rv1 cells (Figure 3I,J, respectively). RAMP1 was used as a control, since it shows an inverse correlation with NKX3.1 mRNA. Similarly, NKX3.1 knockdown led to a more than 2-fold increase in both cell types (Figure 3K,L). These results show that NKX3.1 downregulates LIMK2 mRNA levels. 

We also explored whether NKX3.1 targets LIMK2 protein for degradation. The LIMK2 degradation profile was evaluated in cycloheximide-exposed C4-2 and NKX3.1 shRNA-infected C4-2 cells. NKX3.1 knockdown significantly enhanced LIMK2 stability (Figure 3M,N). Comparable results were observed in 22Rv1 cells, where NKX3.1 knockdown was associated with enhanced LIMK2 stability, thereby confirming that NKX3.1 negatively regulates LIMK2 mRNA and protein (Figure 3O,P). To examine whether NKX3.1 promotes LIMK2 ubiquitylation, C4-2 and 22Rv1 cells were co-infected with NKX3.1 and 6x-His-tagged-ubiquitin, which revealed significantly increased ubiquitylation of LIMK2 upon NKX3.1 overexpression in both C4-2 and 22Rv1 cells (Figure 3Q,R). Figure S3 shows raw data for Figure 3A,C,E,G,M,O,Q,R.

### 2.6. LIMK2 Phosphorylates NKX3.1 at S185 Triggering Its Degradation

**Figure 4:**
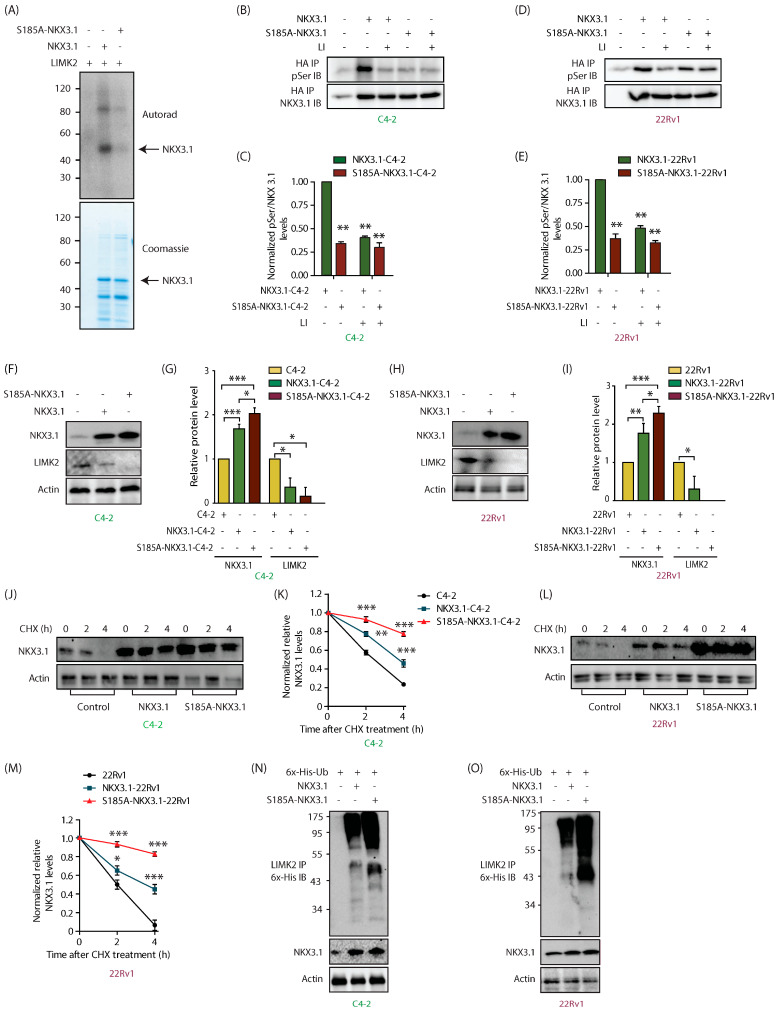
LIMK2 modulates NKX3.1 stability via direct phosphorylation at S185. (**A**) LIMK2 phosphorylates wild-type NKX3.1 but not S185A mutant. In vitro phosphorylation assay was performed using LIMK2, wild type NKX3.1, and S185A-NKX3.1. The top panel represents an autoradiograph, whereas the bottom panel shows the corresponding gel stained with Coomassie blue. In lane 1, LIMK2 was incubated with γ-^32^P-ATP. In lane 2, NKX3.1 was incubated with LIMK2 and γ-^32^P-ATP. In lane 3, S185A-NKX3.1 was incubated with γ-^32^P-ATP and LIMK2. All assays were repeated at least three independent times; (**B**) LIMK2 directly phosphorylates NKX3.1 at S185 in C4-2 cells. Phospho-serine levels were measured in NKX3.1-C4-2 and S185A-NKX3.1-C4-2 cells in response to inhibition of LIMK2 (10 μM LI, 12 h). Ectopically expressed HA-tagged wild type and S185A-NKX3.1 were isolated using HA IP, and the corresponding phospho-Ser levels analyzed; (**C**) Quantification of p-Ser levels relative to NKX3.1 levels obtained from three independent experiments [Mean ± SEM]. All values were normalized with respect to either wild-type or S185A-NKX3.1 overexpressing cells without inhibition [** *p* < 0.01]; (**D**) S185 is the sole site of LIMK2-mediated phosphorylation of NKX3.1 in 22Rv1 cells. 22Rv1, NKX3.1-22Rv1, and S185A-NKX3.1-22Rv1 cells were treated with 10 μM LIMK2 inhibitor (LI) for 12 h after which phospho-Ser and NKX3.1 levels were probed following HA IP. DMSO was used as the vehicle control; (**E**) Quantification of relative p-Ser/NKX3.1 levels in 22Rv1 cells in response to LIMK2 inhibition. The bar graph represents data obtained from three independent experiments, normalized to either NKX3.1-22Rv1 or S185A-NKX3.1-22Rv1 cells without LIMK2 inhibition. These are shown as mean ± SEM with ** *p* < 0.01; (**F**) S185A-NKX3.1 is expressed at a higher level compared to wild-type-NKX3.1. C4-2 cells transiently infected with NKX3.1 and S185A-NKX3.1 retroviruses; (**G**) Quantification of LIMK2 and NKX3.1 protein levels analysis normalized to the actin from three independent experiments. * *p* < 0.05, and *** *p* < 0.001; (**H**) S185A-NKX3.1 is expressed at a higher level compared to wild-type-NKX3.1 in 22Rv1 cells; (**I**) Quantification of data from three independent experiments. * *p* < 0.05, ** *p* < 0.01, *** *p* < 0.001; (**J**) LIMK2-mediated phosphorylation of NKX3.1 at S185 lowers its stability in C4-2 cells. NKX3.1 degradation in C4-2 cells was tested using the cycloheximide assay; (**K**) Quantification of relative NKX3.1 protein levels, normalized to actin, obtained from three independent experiments as described in (**J**). ** *p* < 0.01, and *** *p* < 0.001; (**L**) The phospho-resistant mutant, NKX3.1-S185A, displays higher stability in 22Rv1 cells, as compared to WT. 22Rv1 cells overexpressing NKX3.1 and S185A-NKX3.1 were treated with CHX (20 µg/mL) along with vector-expressing cells. Protein levels were probed at the indicated time points; (**M**) Quantification of relative NKX3.1 protein levels from three independent experiments as described in (**L**). * *p* < 0.05, and *** *p* < 0.001; (**N**) NKX3.1 regulates the ubiquitylation of LIMK2 in C4-2 and (**O**) 22Rv1 cells.

To probe the mechanism further, we analyzed LIMK2-mediated phosphorylation sites on NKX3.1. LIMK2 is a dual-specificity kinase and thus can phosphorylate S, T, or Y. Nevertheless, the only sites reported to be phosphorylated by LIMK2 are serine. The optimal sequence that LIMK2 may prefer also remains unidentified. Based on the cofilin phosphorylation site, we previously predicted that LIMK2 prefers serine residues, which are followed by alanine or glycine, which led to the identification of LIMK2-mediated sites on TWIST1, PTEN, and SPOP in vitro and in cells. Based on these criteria, we identified S185 as the potential site for NKX3.1. We generated S185A phospho-dead mutant and exposed it to a kinase assay with LIMK2. While WT-NKX3.1 was efficiently phosphorylated, the S185A mutant showed no phosphorylation (Figure 4A), confirming that LIMK2 only phosphorylates the S185 site in NKX3.1.

To validate whether the S185 site is indeed phosphorylated by LIMK2 in cells, we isolated HA-tagged WT and S185A-NKX3.1 proteins from the corresponding WT and S185A-NKX3.1-expressing C4-2 cells using HA antibody and analyzed their phospho-serine (phospho-Ser) levels using a phospho-serine antibody. Parental C4-2 cells were used as a negative control. To ensure that equal amounts of WT and mutant protein were analyzed, excess cell lysate was used with a limiting amount of HA antibody. As indicated in Figure 4B,C, WT-NKX3.1 showed >3-fold higher phospho-Ser levels, as compared to the S185A mutant. Importantly, to examine the role of LIMK2 in this process, it was inhibited in both C4-2 and 22Rv1 cells, which caused phospho-Ser levels of NKX3.1 to plummet significantly, whereas phospho-Ser levels of S185A remained unchanged. This occurrence was confirmed in 22Rv1 cells, which too showed that LIMK2 regulates the phosphorylation of NKX3.1 at the S185 site in CRPC cells (Figure 4D,E).

To probe the consequences of NKX3.1 phosphorylation by LIMK2, we ectopically expressed WT and S185A mutant in C4-2 cells, which revealed increased expression of the mutant as compared to WT (Figure 4F,G), indicating that phospho-resistant mutant is more stable than WT. Furthermore, increased NKX3.1 levels were associated with lower levels of LIMK2, confirming the negative loop. Analogous results were observed in 22Rv1 cells (Figure 4H,I). 

To confirm that S185 phosphorylation is responsible for NKX3.1 degradation, protein synthesis was inhibited in C4-2, WT, and S185A-NKX3.1-expressing C4-2 cells using cycloheximide and the degradation pattern of NKX3.1 was evaluated. As indicated, the S185A mutant showed much higher stability as compared to WT (Figure 4J,K). These results were validated in 22Rv1, WT, and S185A-NKX3.1-expressing 22Rv1 cells, which also showed slower degradation of the S185A mutant as compared to the WT protein (Figure 4L,M). 

As S185A-NKX3.1 is more stable than the WT, an equivalent decrease in LIMK2 levels was observed in corresponding C4-2 and 22Rv1 cells (Figure 4F–I). Therefore, we examined the ubiquitylation of LIMK2 in WT and S185A-NKX.1-expressing C4-2 and 22Rv1 cells. As expected, S185A mutant-expressing CRPC cells showed increased ubiquitylation of LIMK2 as compared to WT-NKX3.1-expressing cells, thereby confirming the feedback loop between LIMK2 and NKX3.1 (Figure 4N,O). Figure S4 shows raw data for Figure 4B,D,F,H,J,L,N,O.

### 2.7. LIMK2-Mediated NKX3.1 Phosphorylation Is Critical for Promoting Aggressive Phenotypes

**Figure 5:**
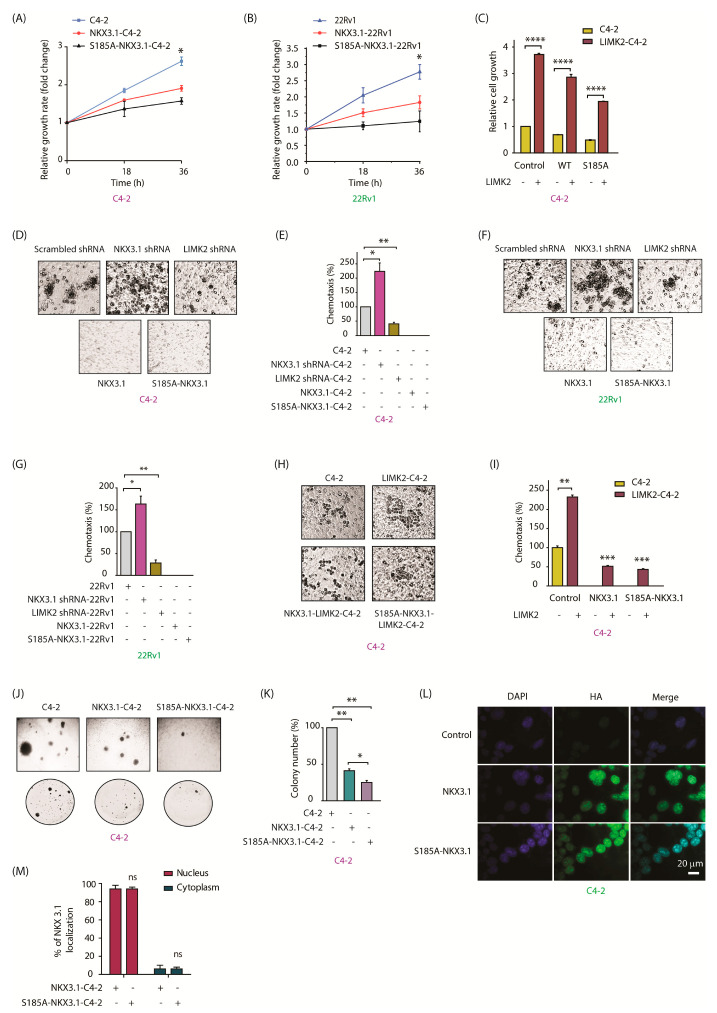
LIMK2-mediated NKX3.1 downregulation promotes oncogenic phenotypes. (**A**) NKX3.1 inhibited cell proliferation in C4-2 and (**B**) 22Rv1 cells. Target cells were seeded in a 24-well plate and infected by NKX3.1 and S185A-NKX3.1 retroviruses for 18 and 36 h. Proliferative activity was measured by MTT assay. The absorbance was measured at 570 nm. The results are plotted as the means ± SD of three independent experiments. * *p* < 0.05; (**C**) C4-2 cells stably expressing NKX3.1 and S185-NKX3.1 were infected by LIMK2 retrovirus. Proliferative activity was measured by MTT assay. All data are from three independent experiments. **** *p* < 0.0001; (**D**) NKX3.1 suppressed cell migration in C4-2 cells. Target cells were infected by NKX3.1 shRNA lentivirus, LIMK2 sRNA lentivirus, NKX3.1 retrovirus, and S185A-NKX3.1 retrovirus. The cells were starved in serum-free media for 12 h and the migration assay was performed using Boyden chambers; (**E**) The results are plotted as the means ± SD of three independent experiments as described in (**D**). * *p* < 0.05, ** *p* < 0.01; (**F**) NKX3.1 suppressed cell migration in 22Rv1 cells; (**G**) The results are plotted as the means ± SD of three independent experiments as described in (**F**). * *p* < 0.05, ** *p* < 0.01; (**H**) Migration assays of C4-2 cells that stably expressed NKX3.1 and S185A-NKX3.1 infected by LIMK2 retrovirus are shown. C4-2 cells were used as control; (**I**) The results are presented as the mean ± SD of three independent experiments. The graph represents the data analysis as compared to untreated C4-2 cells. ** *p* < 0.01, *** *p* < 0.001; (**J**) NKX3.1 and S185A-NKX3.1 inhibit colony formation ability of C4-2 cells; (**K**) Quantitative data analysis of the soft-agar experiment as shown in J. All data were from *n* = 3. * *p* < 0.05, ** *p* < 0.01. (**L**) LIMK2-mediated phosphorylation of NKX3.1 does not impact the subcellular localization of NKX3.1, as both the WT and S185A mutant show nuclear residence. Immunofluorescence micrographs of C4-2 cells infected with WT or S185A mutant and probed with HA antibody (green). Nuclear counterstain is represented by DAPI (blue). [Scale bar = 20 μm]; (**M**) Quantification of subcellular localization based on analysis of the micrographs in (**L**). A total of 100 cells were counted from 20 different frames in each case.

NKX3.1 is a tumor suppressor and inhibits cell proliferation [19]. As phospho-resistant S185A-NKX3.1 is more stable, we reasoned that it should enhance the inhibitory effect of NKX3.1 on cell growth. To test this hypothesis, we measured the relative growth rate of C4-2, NKX3.1-C4-2, and S185A-NKX3.1-C4-2 cells. As expected, S185A expression was more effective in reducing cell proliferation as compared to WT, whereas parental C4-2 cells displayed the maximal growth rate (Figure 5A). Ectopic expression of WT and S185A-NKX3.1 showed a similar pattern in 22Rv1 cells as well, indicating that degradation of NKX3.1 by LIMK2-mediated phosphorylation may be a key step in promoting oncogenic phenotypes (Figure 5B). To investigate this further, we ectopically expressed LIMK2 in C4-2, NKX3.1-C4-2, and S185A-NKX3.1-C4-2 stable cells, and examined the change in cell proliferation rates as compared to the corresponding parental cell lines. LIMK2 overexpression increased the cellular growth in all cell lines, albeit least in S185A-expressing cells, indicating that LIMK2 uses multiple pathways to promote oncogenicity, including NKX3.1 phosphorylation and subsequent degradation (Figure 5C).

We next investigated the effect of NKX3.1 phosphorylation on chemotaxis. As indicated, NKX3.1 knockdown greatly increased cell migration (Figure 5D,E), whereas LIMK2 silencing had the opposite effect. In vitro migration assays in C4-2, NKX3.1-C4-2, and S185A-NKX3.1-C4-2 cells displayed a similar trend, where C4-2 cells showed robust migration, where WT-NKX3.1- and S185A-NKX3.1-expressing cells were impaired in cell migration (Figure 5D,E). We observed an analogous pattern of cellular migration in 22Rv1 cells, where LIMK2 depletion decreased, and NKX3.1 silencing increased cell migration (Figure 5F,G). Consequently, ectopic expression of NKX3.1 (WT and S185A mutant) in 22Rv1 cells significantly inhibited cell migration. We next examined whether LIMK2 overexpression rescues WT and S185A-NKX3.1-mediated suppression of motility in C4-2 cells. While LIMK2 overexpression considerably increased cell motility in C4-2 cells, both WT and S185A-expressing cells showed moderate enhancement, suggesting that NKX3.1 degradation by LIMK2 is an important step in increasing cell migration (Figure 5H,I).

Our previous studies have shown that LIMK2 overexpression strongly induces colony formation [5]. Thus, we performed clonogenic assays to determine the relative cell growth of C4-2, NKX3.1-C4-2, and S185A-NKX3.1-C4-2 stable cell lines under anchorage-independent conditions. Independency from anchorage allows tumor cells to metastasize to other tissues. Parental C4-2 cells showed robust colony formation as expected. In contrast, WT-NKX3.1-C4-2 cells showed significantly fewer colonies and reduced size. S185A-NKX3.1-C4-2 cells were mostly impaired and showed minimal colony formation (Figure 5J,K). These data suggested that S185 phosphorylation is an important mechanism by which LIMK2 regulates NKX3.1 stabilization and functions.

Although our IF data showed that LIMK2 does not regulate the subcellular localization of NKX3.1, nevertheless, we investigated whether phosphorylation-resistant HA-tagged NKX3.1 shows similar localization in C4-2 cells. HA-tagged WT NKX3.1 was used as the control. As shown in Figure 5L,M, both WT and S185A-NKX3.1 showed nuclear localization, confirming that LIMK2 does not affect the nuclear residence of NKX3.1. 

### 2.8. The Phosphomimetic S185D-NKX3.1 Decreases NKX3.1 Stability and Enhances LIMK2 Protein Level by Inhibiting Its Ubiquitylation

**Figure 6:**
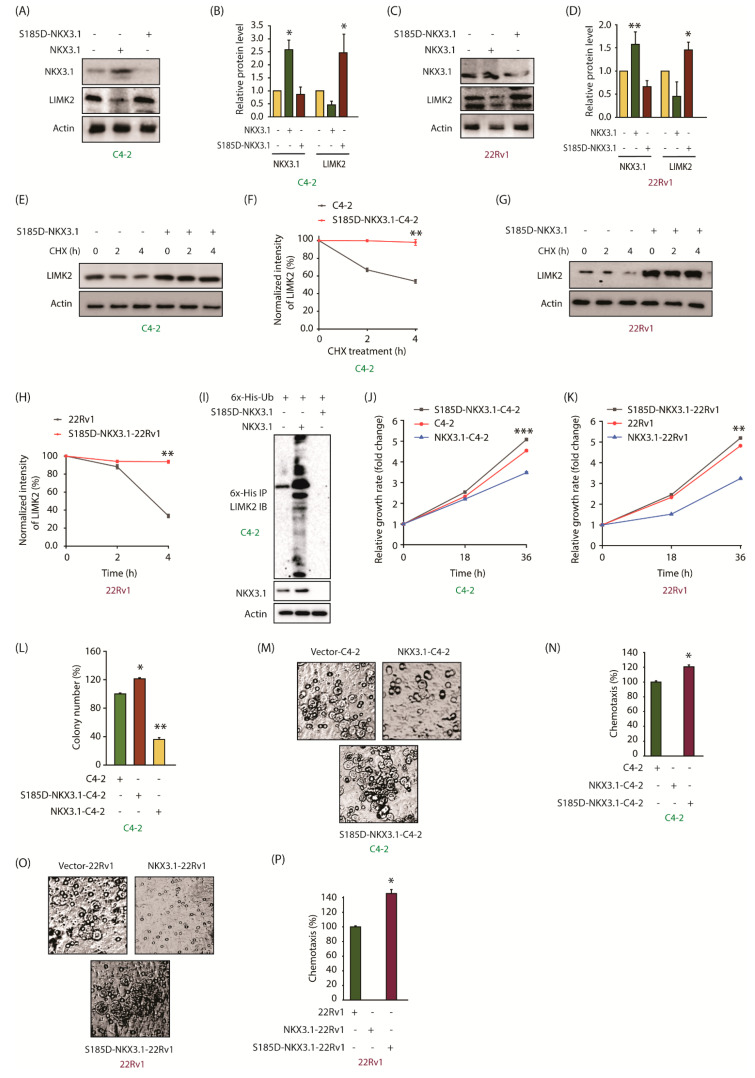
Phospho-mimetic S185D-NKX3.1 is less stable and promotes oncogenic phenotypes. (**A**) S185D-NKX3.1 is expressed at a lower level compared to the wild-type-NKX3.1. C4-2 cells were transiently infected with NKX3.1 and S185D-NKX3.1 retroviruses. Cell lysates were analyzed to detect NKX3.1 and LIMK2 protein levels; (**B**) Quantification of LIMK2 and NKX3.1 levels normalized to actin is shown from three independent experiments. * *p* < 0.05; (**C**) S185D-NKX3.1 is expressed at a lower level compared to the wild type in 22Rv1 cells; (**D**) Quantification of LIMK2 and NKX3.1 levels normalized to actin is shown from three independent experiments. * *p* < 0.05 and ** *p* < 0.01; (**E**) The stability of LIMK2 is increased due to S185D mutation in NKX3.1. LIMK2 degradation in C4-2 cells was tested using CHX; (**F**) Quantification of LIMK2 levels normalized to actin is shown from three independent experiments. ** *p* < 0.01; (**G**) Stability of LIMK2 increases in S185D-NKX3.1-22Rv1 cells; (**H**) Quantification of LIMK2 levels normalized to actin is shown from three independent experiments. ** *p* < 0.01; (**I**) S185D expression in C4-2 cells inhibits the ubiquitylation of LIMK2; (**J**) Cell viability was measured at indicated times in C4-2 and (**K**) 22Rv1 cells treated with vector, NKX3.1 and S185D-NKX3.1 retroviruses. Target cells were seeded in a 24-well plate and infected using vector, NKX3.1 and S185D-NKX3.1 retroviruses. All data are from three independent experiments. ** *p* < 0.01 and *** *p* < 0.001; (**L**) NKX3.1 suppressed colony formation in C4-2 cells. Stable C4-2 cells expressing either NKX3.1 or S185D-NKX3.1 were used for colony formation assay. The colony formation data are plotted as the means ± SD of three independent experiments. * *p* < 0.05 and ** *p* < 0.01; (**M**) While WT NKX3.1 suppressed cell migration, S185D-NKX3.1 increased it in C4-2 cells; (**N**) The chemotaxis results in C4-2 cells are plotted as the means ± SD of three independent experiments. * *p* < 0.05; (**O**) While WT NKX3.1 suppressed cell migration, S185D-NKX3.1 increased it in 22Rv1 cells; (**P**) The chemotaxis results obtained from 22Rv1 cells are plotted as the means ± SD of three independent experiments. * *p* < 0.05.

As phospho-resistant mutation at the S185 site increases the stability of NKX3.1, we generated the corresponding phospho-mimetic mutant (S185D) to examine whether it exhibits decreased stability as compared to WT. WT and S185D-NKX3.1 were transiently expressed in C4-2 cells using vector-infected cells as a control. WT NKX3.1 showed robust expression as expected (Figure 6A,B). In contrast, the S185D mutant showed minimal expression, which was even less than endogenous NKX3.1 levels. Similarly, S185D-NKX3.1 levels were much lower in 22Rv1 cells as compared to WT NKX3.1 (Figure 6C,D). A reduction in NKX3.1 levels in C4-2 and 22Rv1 cells was simultaneously associated with a corresponding increase in LIMK2 levels, signifying the negative feedback loop. To confirm this crosstalk, we determined the stability of LIMK2 in C4-2 and S185D-C4-2 cells using cycloheximide. LIMK2 was significantly more stable in S185D mutant-expressing cells than C4-2 cells (Figure 6E,F). LIMK2 was also more stable in S185D-22Rv1 cells as compared to vector-infected 22Rv1 cells (Figure 6G,H). Finally, LIMK2 ubiquitylation was analyzed in 6x-His-ubiquitin-expressing C4-2, NKX3.1-C4-2, and S185D-NKX3.1-C4-2 cells. LIMK2 was isolated from the aforementioned cells and its ubiquitylation analyzed using 6x-His antibody. While WT NKX3.1 induced robust ubiquitylation, S185D expression resulted in no ubiquitylation (Figure 6I). These results using the phosphomimetic mutant thus confirm the negative relationship between LIMK2 and NKX3.1 in CRPC cells.

### 2.9. The Phosphomimetic S185D-NKX3.1 Promotes Cell Proliferation, Colony Formation, and Chemotaxis

As our data showed that the S185D mutant is expressed at a lower level as compared to WT-NKX3.1, we measured its impact on cell proliferation and chemotaxis. In accordance with its decreased stability, S185D-NKX3.1-expressing C4-2 cells showed increased proliferative capacity, compared to parental cells, whereas WT-expressing cells showed a reduced proliferation rate (Figure 6J). We observed a similar trend in 22Rv1 cells upon ectopic expression of S185D mutant (Figure 6K). Similarly, S185D mutant promoted colony formation, whereas WT inhibited it (Figure 6L). Likewise, ectopic expression of NKX3.1 fully inhibited chemotaxis in both C4-2 and 22Rv1 cells, whereas S185D expression promoted cell motility, which was higher than the corresponding parental cells (Figure 6M–P). These results are consistent with the increased stability of LIMK2 in S185D-expressing CRPC cells (Figure 6E–H), which promotes oncogenic phenotypes. Figure S6 shows raw data for Figure 6A,C,E,G,I.

### 2.10. NKX3.1 Downregulates AKT Activation and Decreases AR and ARv7 Levels in CRPC Cells

**Figure 7:**
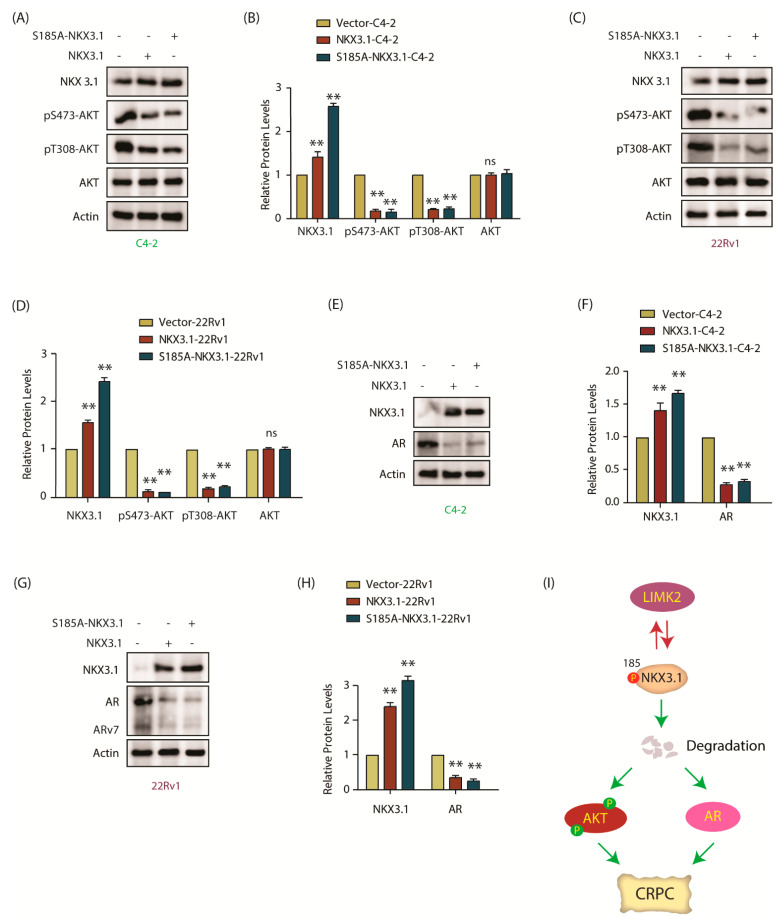
NKX 3.1 inhibits AKT phosphorylation, and depletes AR and ARv7. (**A**) Levels of phospho-AKT in NKX3.1- and S185A-NKX3.1-overexpressing C4-2 cells are much lower than vector-expressing control cells. Control, NKX3.1-C4-2, and S185A-NKX3.1-C4-2 cells were assayed for p-AKT levels along with AKT and actin; (**B**) The histogram shows changes in AKT phosphorylation levels in response to NKX3.1 and S185A-NKX3.1 expression. The data obtained from three independent experiments were normalized against actin and represented as mean ±SEM [** *p* < 0.01, ns = not significant]; (**C**) The extent of AKT phosphorylation is lowered by ectopic overexpression of wild-type and phospho-resistant NKX3.1 in 22Rv1 cells. p-AKT and AKT levels in vector control, NKX3.1-22Rv1, and S185A-NKX3.1-22Rv1 cells were monitored using Western blot analysis; (**D**) Quantification of AKT phosphorylation levels obtained from three independent experiments as depicted in C. [** *p* < 0.01, ns = not significant]; (**E**) Both wild-type NKX3.1 and S185A-NKX3.1 deplete AR protein levels in C4-2 cells. The effect of ectopic expression of wild-type and phospho-resistant NKX3.1 on AR protein levels was examined using Western blot; (**F**) The bar graph shows change in AR and NKX3.1 protein levels normalized against actin. The data from three independent experiments were plotted with ** *p* < 0.01 compared to vector-expressing cells; (**G**) Ectopic expression of NKX3.1 and S185A-NKX3.1 decreases AR and ARv7 protein levels in 22Rv1 cells. Vector-expressing control, wild-type NKX3.1, and phospho-resistant NKX3.1-overexpressing 22Rv1 cells were lysed and probed for AR and ARv7 protein levels using Western blot analysis; (**H**) The bar graph shows changes in AR and ARv7 protein levels in 22Rv1, NKX3.1-22Rv1, and S185A-NKX3.1-22Rv1 cells. The data from three independent experiments were plotted as mean ± SEM, ** *p* < 0.01 vs. 22Rv1 vector-expressing control cells; (**I**) Schematic model portraying the plausible role of LIMK2-NKX3.1 signaling in CRPC pathogenesis.

As NKX3.1 is known to inhibit AKT activation, we investigated whether LIMK2-mediated phosphorylation of NKX3.1 regulates AKT activation. While parental C4-2 cells showed robust AKT activation as visualized by S473 and T308 phosphorylation, ectopic expression of WT or S185A mutant fully suppressed it, whereas total AKT levels remained unchanged in both C4-2 and 22Rv1 cells (Figure 7A–D). AR levels were next analyzed in C4-2, NKX3.1, and S185A-NKX3.1 cells, which also showed severely reduced AR levels upon WT or S185A expression (Figure 7E,F). In 22Rv1 cells, both AR and ARv7 levels decreased drastically upon WT and mutant NKX3.1 expression (Figure 7G,H). Notably, in each case, S185A-NKX3.1 was expressed at higher levels as compared to WT; nevertheless, we did not observe any differences in AR and ARv7 levels between the two cell types. It was presumably because WT NKX3.1 expression decreased AR and ARv7 to almost basal levels, therefore a relatively higher expression of mutant NKX3.1 could not show any additional impact. Figure S7 shows raw data for Figure 7A,C,E,G.

### 2.11. NKX3.1 Suppresses Tumor Growth In Vivo

Our results indicated that both WT and S185A-NKX3.1 suppress aggressive phenotypes in cells, but the latter is more effective, leading us to hypothesize that while WT NKX3.1 may partially inhibit tumorigenesis in mice, S185A mutant should fully inhibit it. Therefore, we investigated the tumor-suppressing potential of C4-2 and WT NKX3.1-C4-2 cells in nude mice. C4-2 and NKX3.1-C4-2 cells were injected in the left and right flanks, respectively. We measured tumor size every alternative day. As expected, animals receiving C4-2 developed robust tumors. By contrast, WT-NKX3.1 expression fully suppressed tumorigenesis (Material Figure S8A,B). As WT-NKX3.1 completely suppressed tumorigenesis, S185A mutant was not analyzed in xenograft models. These results thus highlight the tumor-suppressive impact of NKX3.1 in CRPC. 

## 3. Discussion

NKX3.1, an androgen-regulated homeobox transcription factor, was the first known prostate epithelium-specific marker, which is indispensable for normal prostate and testes development [26]. NKX3.1 promotes prostatic epithelial specification and differentiation and is essential for maintaining luminal stem cells [14,28]. NKX3.1 is also an established marker for PCa. NKX3.1 is located on the short arm of chromosome 8p21. Loss of heterozygosity (LOH) at this locus occurs in up to 89% of high-grade PIN lesions and up to 86% of prostate adenocarcinomas [29]. Thus, NKX3.1 is present in primary prostate adenocarcinomas, although the levels decline in higher-grade lesions but are still not lost completely, consistent with its haploid status [18,30]. Nevertheless, the loss or reduction in NKX3.1 levels is a key initiating event in PCa [31,32]. Germline loss-of-function of Nkx3.1 in mice causes pre-malignant lesions similar to PIN. NKX3.1 gene copy loss is significantly more frequent in CRPC than in localized disease, indicating that a loss or reduction in NKX3.1 levels is critical for PCa progression, including in CRPC.

Although NKX3.1 transcriptional regulation certainly reduces its levels during PCa progression in some cases, there is a clear disagreement between NKX3.1 protein and mRNA levels in normal and PCa clinical tissues [30]. NKX3.1 protein was reduced in four out of six specimens, which had either normal or increased mRNA levels [30]. Similarly, NKX3.1 copy number and NKX3.1 mRNA levels do not show a correlation [33,34,35]. These findings sparked intense interest in examining its regulation at a post-translational level, which revealed it to be a target of several different kinases. 

NKX3.1 is phosphorylated by casein kinase-2 (CK2) at T89 and T93, which stabilizes NKX3.1 [36]. Similarly, PIM1 phosphorylates NKX3.1 at both S185 and S186, which increases its stability [37]. In contrast, DYRK1 phosphorylates only at S185, which destabilizes NKX3.1 [38]. Thus, it appears that solo phosphorylation at S185 degrades NKX3.1, but simultaneous or independent phosphorylation at S186 stabilizes NKX3.1. NKX3.1 is phosphorylated at S196 and subsequently degraded in response to TNF-α and IL-1b treatment in PCa cells; however, the kinase identity remains unknown [39]. Similarly, active ATM phosphorylates NKX3.1 at residues T134 and T166, triggering NKX3.1 degradation during DNA damage [40]. Thus, NKX3.1 levels play a crucial role in the DNA damage response (DDR) [41]. Protein kinase C (PKC) phosphorylates NKX3.1 at S48; however, the exact role of this event in NKX3.1 functions or stability remains unknown [42]. Thus, modulating the protein stability of NKX3.1 is a critical mechanism both under physiological and pathological conditions.

Our recent studies uncovered LIMK2 as a clinical target for CRPC [4]. We showed that LIMK2 stabilizes TWIST1 by direct phosphorylation at four sites, which leads to EMT and cancer stem cell phenotypes in CRPC [4]. LIMK2 degrades tumor suppressor SPOP by direct phosphorylation, which in turn stabilizes AR, ARv7, and c-Myc, promoting CRPC. Phospho-dead SPOP fully abrogates tumorigenesis in mice, demonstrating that SPOP downregulation by LIMK2 is a key event in CRPC [9]. More recently, we have shown that LIMK2 degrades PTEN and inhibits its phosphatase activity by direct phosphorylation at five sites [10]. PTEN destabilizes LIMK2 in response, and thus LIMK2 levels were significantly more in PTEN^−/−^ prostates as compared to prostates from PTEN^+/+^ mice. PTEN loss often occurs in response to hypoxia. We further observed that hypoxia increases LIMK2 levels, which in turn results in PTEN degradation in CRPC.

Importantly, PTEN and NKX3.1 are intricately linked to PCa progression. While NKX3.1 LOH occurs at an early stage of PCa progression, which leads to PIN [43], PTEN loss occurs at a relatively advanced stage. Nevertheless, loss of Nkx3.1 and Pten acts synergistically, leading to increased Akt signaling and high-grade PIN lesions [44]. More recently, PTEN was shown to dephosphorylate NKX3.1 at S185, which stabilizes it. Thus, PTEN loss also promotes a decrease in NKX3.1 levels [45]. 

This study uncovered a new mechanism of NKX3.1 regulation by LIMK2 in CRPC pathogenesis. LIMK2 directly phosphorylates NKX3.1, which results in its degradation. Consequently, S185A-NKX3.1 is more effective in resisting cell growth, cell migration, and anchorage-independent growth. Interestingly, LIMK2 is often regulated by its substrates, which prompted us to investigate whether NKX3.1 controls LIMK2. Ectopic expression of WT or S185A mutant decreased LIMK2 levels by increased ubiquitylation, confirming the feedback loop. Importantly, LIMK2 also robustly downregulates NKX3.1 mRNA levels, although the mechanism remains unclear.

As many studies have documented that PTEN loss causes NKX3.1 downregulation, our findings provide a new link between NKX3.1 and PTEN via LIMK2. We show that LIMK2 downregulates both PTEN and NKX3.1 by direct phosphorylation. Thus, if NKX3.1 is genomically lost, it increases LIMK2 levels due to increased stabilization, causing PTEN downregulation by LIMK2. PTEN loss also increases LIMK2 levels, which in turn can degrade NKX3.1 (Figure 7I). 

## 4. Materials and Methods

### 4.1. Cell Lines, Antibodies, and Chemicals

C4-2, HEK293T, 22Rv1, and Phoenix cells were purchased from ATCC. Each antibody was used at 1-1000 dilution. Antibodies details, including their vendors and RRID number, are provided in Table S1. Cycloheximide, MG132, and polybrene were purchased from Sigma (Sigma-Aldrich, St. Louis, MO, USA). LIMK2 inhibitor was synthesized according to the published protocol.

### 4.2. LIMK2 and NKX3.1 shRNAs

The sequences of LIMK2 shRNAs were shown in our previous studies [5]. NKX3.1 shRNAs were cloned in pLKO.1 vector [46]. NKX3.1 shRNA sequences are provided in Table S2. Scrambled, LIMK2, and NKX3.1 shRNA lentiviruses were generated and used to infect C4-2 and 22Rv1 cells as before [12]. Puromycin (2 µg/mL) selection was used to generate stable cell lines.

### 4.3. Site-Directed Mutagenesis, Expression, and Purification of LIMK2, Wild-Type (WT), and S185A-NKX3.1 Mutant

Full-length human NKX3.1 was cloned into a pTAT-HA [47], which possesses an N-terminal 6x-His-tag using BamHI and XhoI sites. Using site-directed mutagenesis, we generated S185A and S185D mutants of NKX3.1. HA-tagged WT, S185A-NKX3.1 and S185D NKX3.1 mutants were cloned into VIP3 mammalian retroviral vector. WT and mutant NKX3.1 were produced in *E. coli* and purified using Ni-NTA beads as before [48]. LIMK2 was generated in SF9 cells as we reported before [5].

### 4.4. In Vitro Kinase Assays

Recombinant LIMK2 on Ni-NTA beads was treated with 1 mM ATP in kinase buffer for 2 h as reported earlier [49]. Subsequently, the beads were washed 3 times with kinase buffer to remove all excess ATP. LIMK2 was eluted using imidazole buffer and incubated with recombinant NKX3.1 (WT and mutant) in kinase buffer along with and 1–5 µCi [γ-^32^P-ATP] for 20–30 min at RT. The reaction was stopped by the addition of SDS-PAGE dye followed by SDS-PAGE gel. The resulting radioactivity was determined by autoradiography.

### 4.5. Transfection and Retroviral Infection

To generate WT or S185A-NKX3.1 retrovirus, the corresponding plasmids were transfected into Phoenix cells using calcium phosphate [12]. The supernatants containing virus particles were collected 48 h post-transfection and used to infect the target cells (C4-2 and 22Rv1) in the presence of polybrene. Protein expression was analyzed 32 h post-infection. Stable cells were generated using puromycin.

### 4.6. Western Blotting

First, 2 × 10^6^ cells were plated in 100-mm dishes. After 12 h, they were infected with retroviruses or shRNA lentiviruses. After 32–36 h post-infection, cells were rinsed twice with chilled phosphate-buffered saline and lysed in modified RIPA (1% NP-40, 20 mM Tris, pH 7.5, 150 mM NaCl, 2 mM EDTA, 0.25% sodium deoxycholate) buffer, supplemented with protease inhibitors. After 30 min on ice, cell lysate was centrifuged at 10,000 rpm for 10 min at 4 °C. Bradford assay was used for protein quantification. The proteins were separated using SDS-PAGE and transferred to a PVDF membrane. The blocking was done using 5% skim milk in TBST (20 mM Tris, pH 7.4, 150 mM NaCl, and 0.1% Tween 20). The membrane was incubated with a primary antibody overnight followed by 3–4 h treatment with the secondary antibody. The protein was detected using chemiluminescence-based detection. Image J program was used for densitometry analysis.

### 4.7. Isolation of Cytosolic and Nuclear Fractions

C4-2 and 22Rv1 cells were washed with ice-cold PBS, resuspended in buffer A (10 mM Tris, pH 7.9, 10 mM KCl, 1.5 mM MgCl_2_, 0.5 mM DTT, 0.05% NP40, and 1 mM PMSF), and incubated on ice for 10 min. The lysates were centrifuged at 3000 rpm for 10 min at 4 °C. To separate the nuclear fraction, the pellet was resuspended in buffer B (5 mM Tris, pH 7.9, 1.5 mM MgCl_2_, 0.2 mM EDTA, 0.5 mM DTT, 26% glycerol (*v/v*), 300 mM NaCl, and 1 mM PMSF), and homogenized by passing 10 times through a 27½-gauge needle. The lysates were incubated on ice for 30 min and the nuclear fraction was separated by centrifugation at 24,000× *g* at 4 °C for 20 min. The cytosolic and nuclear extracts were further analyzed by Western blotting [50]. 

### 4.8. Cell Viability Assay 

Cells were seeded in triplicate in a 24-well plate at 1000 cells per well. After 12 h, the cells were infected with the virus. To detect cell viability, 25 µL of 3-(4,5-dimethylthiazol-2-yl)-2,5-diphenyl tetrazolium bromide (MTT), at a final concentration of 0.5 mg/m, were added to each well at indicated times. After 4 h of incubation at 37 °C, the medium was replaced with 500 µL of DMSO. Absorbance was read at 570 nm using a microplate reader (Tecan Spark multimode).

### 4.9. Dose-Response Assay with LIMK2 Inhibitor

C4-2 and 22Rv1 cells were seeded into 96-well plates at 10^4^ cells/well concentration. Then, 12 h after seeding, LIMK2 inhibitor was added to the cells at different concentrations (10 nM, 100 nM, 1 µM, 10 µM, and 100 µM) to determine the IC_50_ value. An equal volume of DMSO was used as the control. After 48 h of incubation, cell viability was determined using MTT assay. Data were analyzed by GraphPad Prism 5 Software (Graphpad, CA, USA). All experiments were conducted in triplicate, two independent times.

### 4.10. Clonogenic Assay

Soft agar assay was conducted as before (21). Briefly, 0.5% agar was made by mixing an equal amount of Noble agar (1%; DNA grade) and 2 × RPMI (20% FBS) and plated in a 24-well plate. Cells (1250 per well) were mixed with 500 µL of 0.7% agarose in 2 × RPMI 1640 (20% FBS) and added on top of solidified base agar. In total, 500 µL of growth medium were added per well. The cells were fed every 3 days with a culture medium. After 3–4 weeks of incubation at 37 °C, colonies were stained with 0.01% crystal violet and incubated at RT for 45 min. Colonies were detected by a light phase-contrast microscope.

### 4.11. Migration Assay

Migration assay was conducted as we reported before (22). CRPC cells were serum-starved for 12 h in serum-free media and recovered by limited trypsin digestion. Then, 10^5^ cells were suspended in 300 μL of DMEM supplemented with 0.5% bovine serum albumin and added to the top compartment of a Boyden chamber, and 400 μL of full growth media were added to the bottom compartment. After incubating for 4 h at 37 °C, chambers were taken out and the inner surface of the membrane was wiped with a cotton applicator. Chambers were washed once with PBS, and cells were counted under a phase-contrast microscope in 10 random fields at 100× magnification. The assays were performed in triplicate, four times. To allow for comparison between multiple assays, the data were normalized and expressed as a percentage of the number of cells present on the membrane.

### 4.12. Ubiquitylation Assay

For ubiquitylation assay, cells were coinfected with 6x-His-ubiquitin (cloned into VIP3 retroviral vector) and NKX3.1 (wild type or S185A-NKX3.1) retroviruses to detect LIMK2 ubiquitylation. Alternatively, cells were coinfected with 6x-His-ubiquitin and LIMK2 retroviruses to detect NKX3.1 ubiquitylation. After 24 h, the cells were treated with MG132 (Sigma) for an extra 12 h. Following cell lysis, ubiquitylated proteins were isolated using the corresponding antibody, followed by 6x-His IB.

### 4.13. LIMK2 Inhibitor

N-benzyl-N-ethyl-4-(N-phenylsulfamoyl) benzamide (LIMK2 inhibitor) was synthesized as before [25]. This inhibitor denoted as LI in figures was used at the 10 μM concentration for 12 h.

### 4.14. qPCR Assay

qPCR assays were conducted according to our previously published methods [4]. The primer sequences used for qPCR are included in Table S3.

### 4.15. C4-2 Xenografts in Nude Mice

Male NCRNU-M athymic nude mice (4 weeks old) were obtained from Taconic Laboratories. All mice were housed at the Purdue animal facility, which provided husbandry and clinical care. Tumor injections were done as reported previously [4]. Briefly, 1 × 10^6^ cells were mixed with 50% Matrigel and injected subcutaneously in nude mice. Tumor measurements were done every alternative day. Tumor diameter was measured using vernier calipers and volume was calculated based on the formula for spheroid (volume = 4πr^3^/3, where r is the radius).

### 4.16. Statistical Analysis

All data are presented as the mean and error bars represent standard deviation (SD) from 3 biological replicates. Statistical analyses were performed using OriginPro 2019. The significance of difference was determined by one-way analysis of variance followed by Bonferroni’s post hoc test. *p* < 0.05 was considered statistically significant.

## 5. Conclusions

While NKX3.1 homozygously deleted tumors can only be treated by gene therapy, preserving the levels of NKX3.1 in heterozygous tumors by LIMK2 inhibition provides a tremendous opportunity for developing targeted therapy in CRPC. Further, as NKX3.1 downregulates AR transcription and inhibits AKT signaling, restoring its levels by inhibiting LIMK2 is expected to be especially beneficial by co-targeting two driver pathways in tandem, a highly desirable requisite for developing effective PCa therapeutics. 

## Data Availability

Not applicable.

## Supplementary Materials

The following are available online at https://www.mdpi.com/article/10.3390/cancers13102324/s1. Figure S1: Full-length immunoblot images of Figure 1; Figure S2: Full-length immunoblot images of Figure 2; Figure S3: Full-length immunoblot images of Figure 3; Figure S4: Full-length immunoblot images of Figure 4; Figure S5: Dose-response curve of LIMK2 inhibitor in C4-2 and 22Rv1 cells. Figure S6: Full-length immunoblot images of Figure 6; Figure S7: Full-length immunoblot images of Figure 7; Figure S8: In vivo data in nude mice. Table S1: List of antibodies; Table S2: Sequence of NKX3.1 shRNA; Table S3: Details of real-time qPCR primers.
