# Correction: Sooreshjani et al. LIMK2-NKX3.1 Engagement Promotes Castration-Resistant Prostate Cancer. *Cancers* 2021, *13*, 2324

**DOI:** 10.3390/cancers18081274

**Published:** 2026-04-17

**Authors:** Moloud A. Sooreshjani, Kumar Nikhil, Mohini Kamra, Dung N. Nguyen, Dinesh Kumar, Kavita Shah

**Affiliations:** Department of Chemistry and Purdue University Center for Cancer Research, Purdue University, 560 Oval Drive, West Lafayette, IN 47907, USA; maflakis@purdue.edu (M.A.S.); niksbiotech@gmail.com (K.N.); nguye445@purdue.edu (D.N.N.); kumar516@purdue.edu (D.K.)

## Error in Figure

In the original publication [1], there was a mistake in Figures S2G, S4J,L and 4J,L as published. Figure S2G had the incorrect actin blot. The background of Figure 4J,L were altered. The corrected Figures S2G and S4J,L appears below. The authors state that the scientific conclusions are unaffected. This correction was approved by the Academic Editor. The original publication has also been updated.

Figure 4:

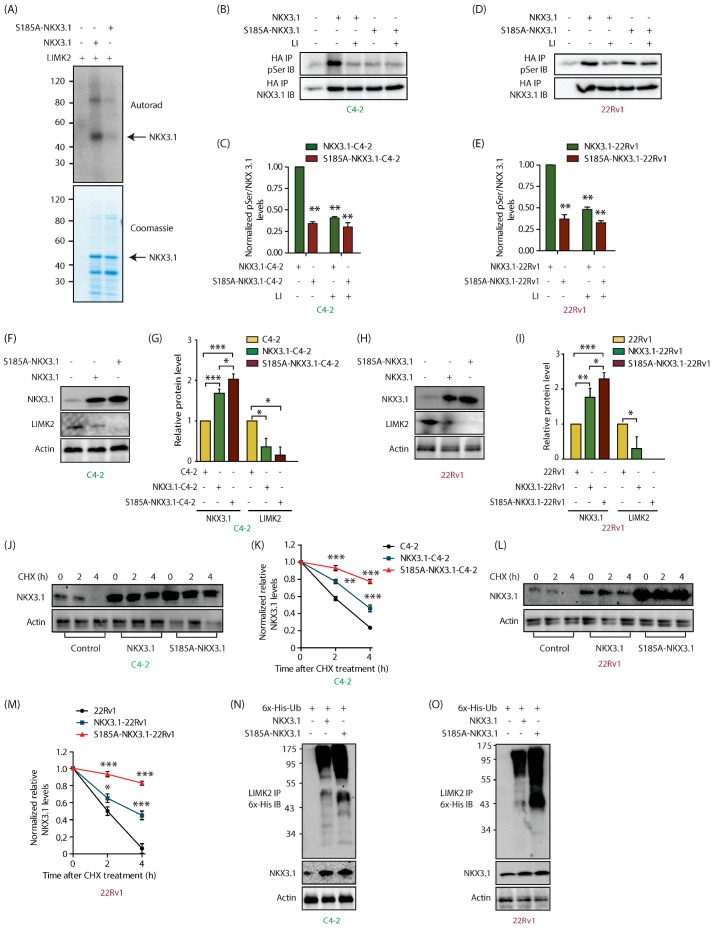


Figure S2:

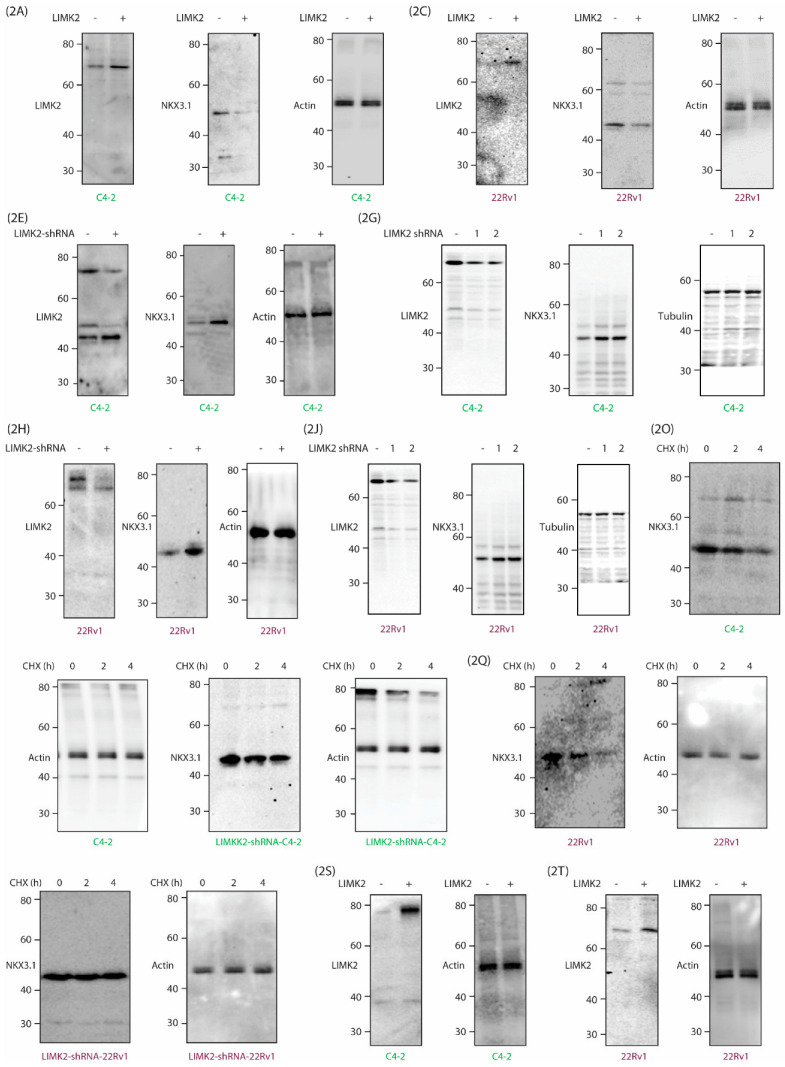


Figure S4:

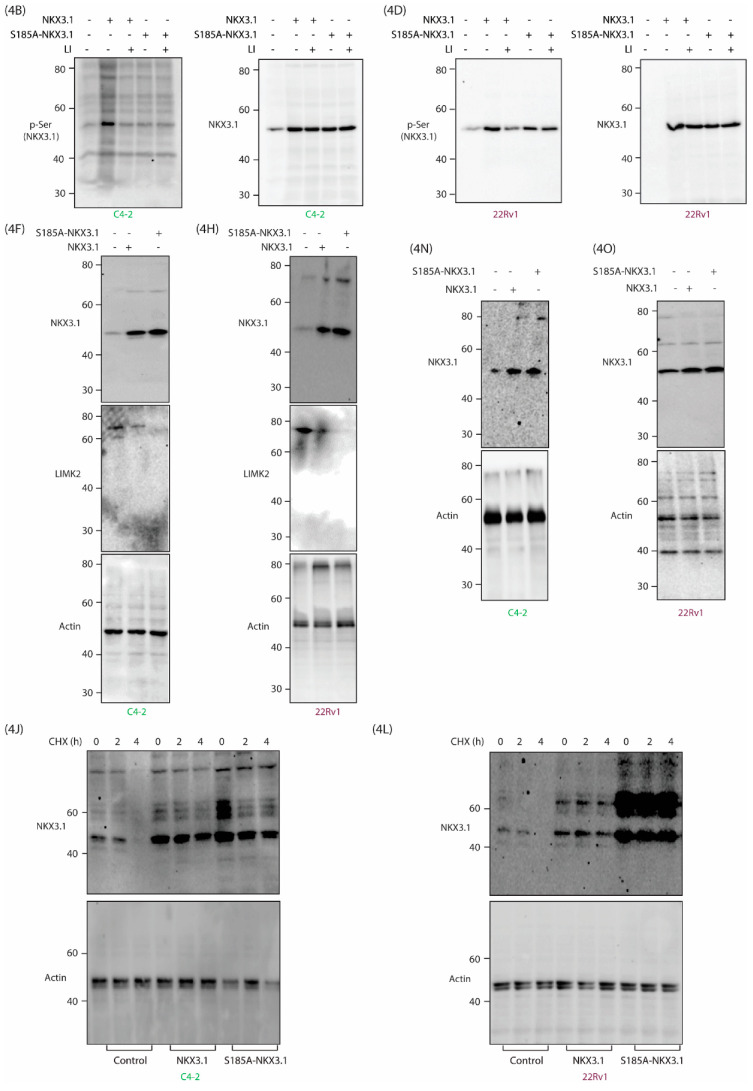


## References

[1] Sooreshjani M.A., Nikhil K., Kamra M., Nguyen D.N., Kumar D., Shah K. (2021). LIMK2-NKX3.1 Engagement Promotes Castration-Resistant Prostate Cancer. Cancers.

