# Supplementary material for: LIMK2-NKX3.1 Engagement Promotes Castration-Resistant Prostate Cancer
Source: Cancers (Basel). 2021 May 12;13(10):2324. doi: 10.3390/cancers13102324 (PMC8151535; doi:10.3390/cancers13102324)
Supplement: Supplementary file 1 [file cancers-13-02324-s001.zip › cancers-1173792- supplementary-figures.pdf]

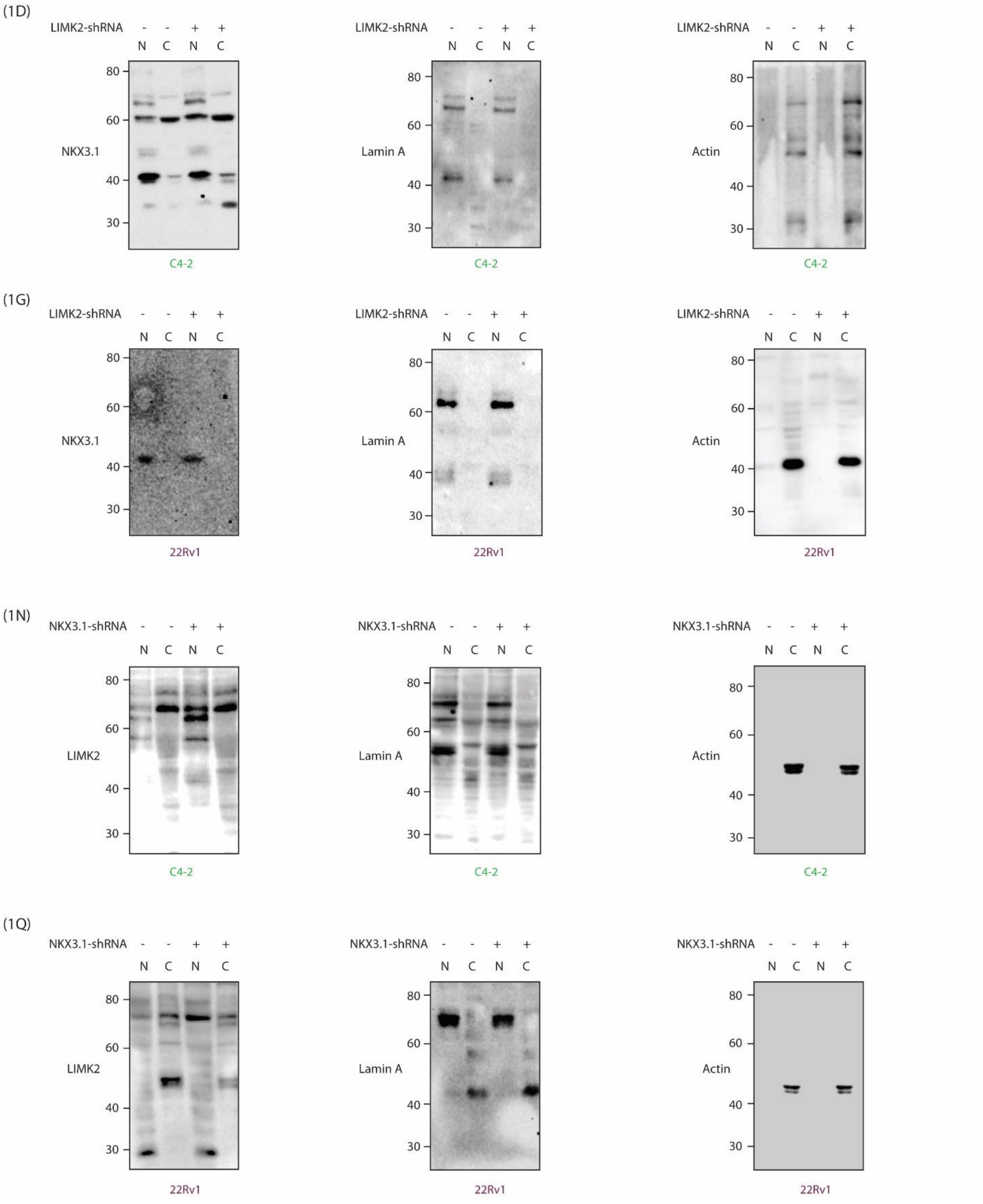

(1D)
LIMK2-shRNA - - + +
N C N C
NKX3.1
Lamin A
Actin
80
60
40
30
C4-2
(1G)
LIMK2-shRNA - - + +
N C N C
NKX3.1
Lamin A
Actin
22Rv1
(1N)
NKX3.1-shRNA - - + +
N C N C
LIMK2
Lamin A
Actin
C4-2
(1Q)
NKX3.1-shRNA - - + +
N C N C
LIMK2
Lamin A
Actin
22Rv1

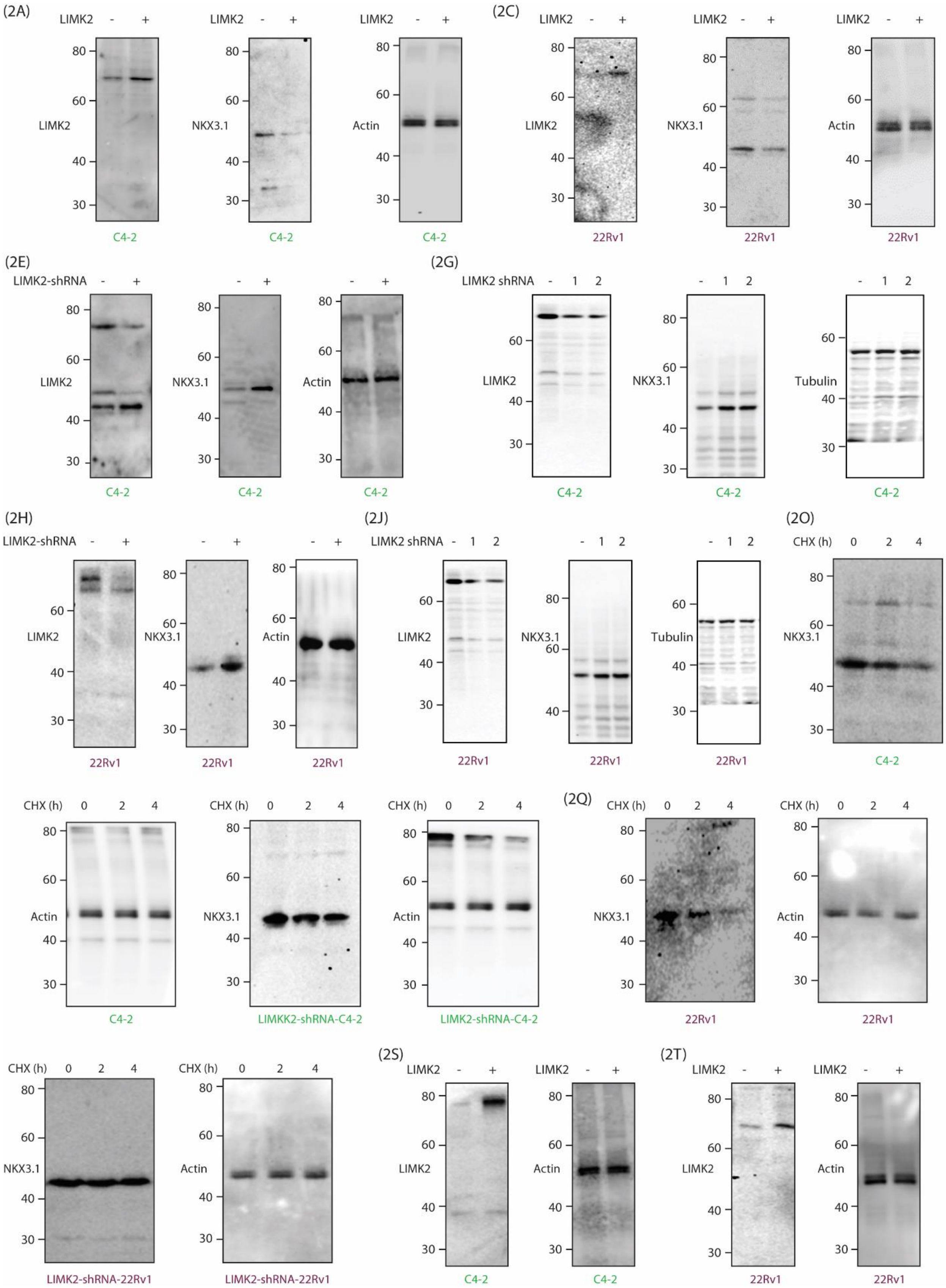
(2A)
LIMK2 - +
LIMK2
NKX3.1
Actin
C4-2
(2C)
LIMK2 - +
LIMK2
NKX3.1
Actin
22Rv1
(2E)
LIMK2-shRNA - +
LIMK2
NKX3.1
Actin
C4-2
(2G)
LIMK2 shRNA - 1 2
LIMK2
NKX3.1
Tubulin
C4-2
(2H)
LIMK2-shRNA - +
LIMK2
NKX3.1
Actin
22Rv1
(2J)
LIMK2 shRNA - 1 2
LIMK2
NKX3.1
Tubulin
22Rv1
(2O)
CHX (h) 0 2 4
NKX3.1
C4-2
Actin
C4-2
NKX3.1
LIMKK2-shRNA-C4-2
Actin
LIMK2-shRNA-C4-2
(2Q)
CHX (h) 0 2 4
NKX3.1
22Rv1
Actin
22Rv1
NKX3.1
LIMK2-shRNA-22Rv1
Actin
LIMK2-shRNA-22Rv1
(2S)
LIMK2 - +
LIMK2
Actin
C4-2
(2T)
LIMK2 - +
LIMK2
Actin
22Rv1
80
60
40
30

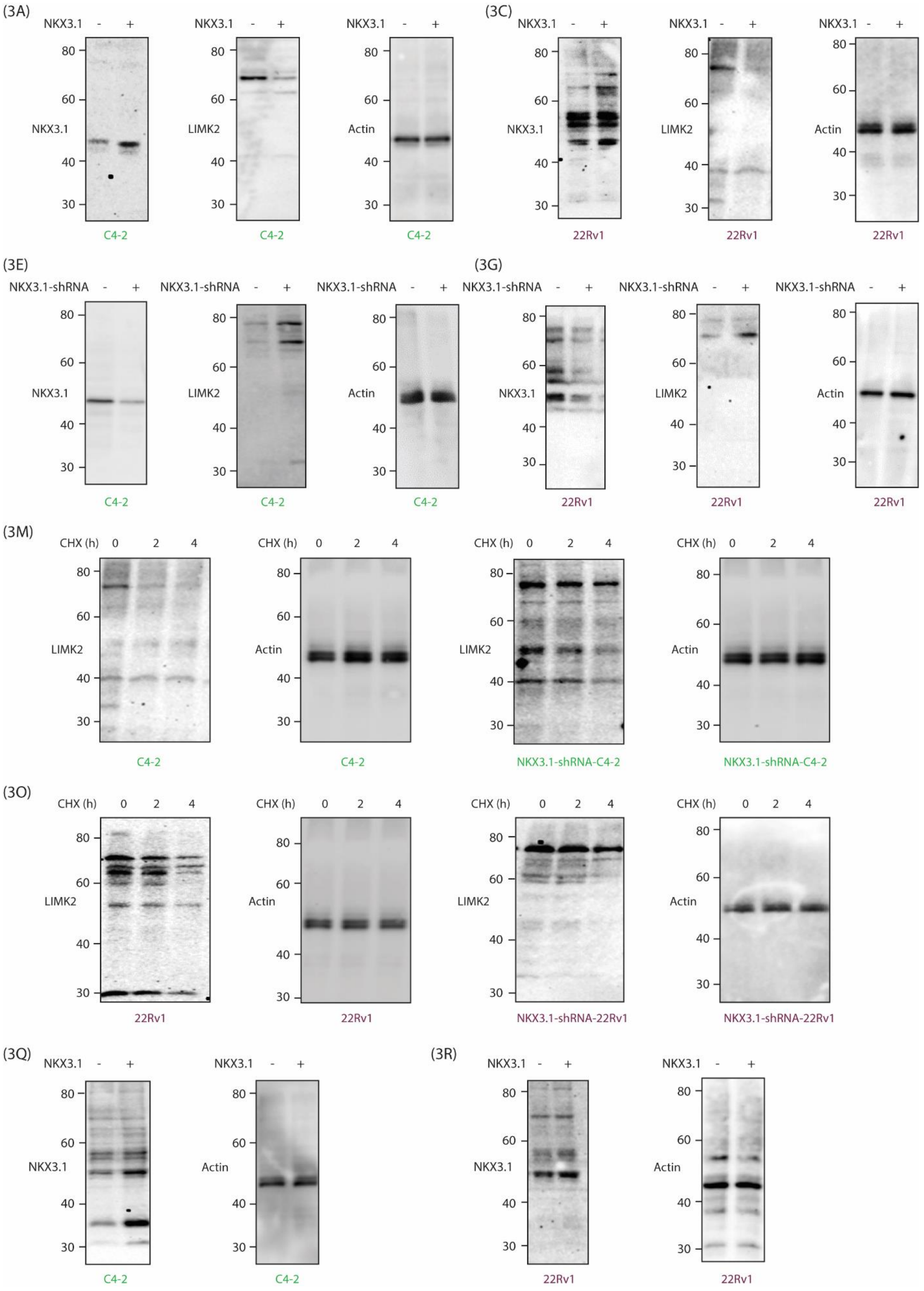
(3A)
NKX3.1 - +
80
60
40
30
NKX3.1
LIMK2
Actin
C4-2
(3C)
22Rv1
(3E)
NKX3.1-shRNA - +
(3G)
(3M)
CHX (h) 0 2 4
NKX3.1-shRNA-C4-2
(3O)
NKX3.1-shRNA-22Rv1
(3Q)
(3R)

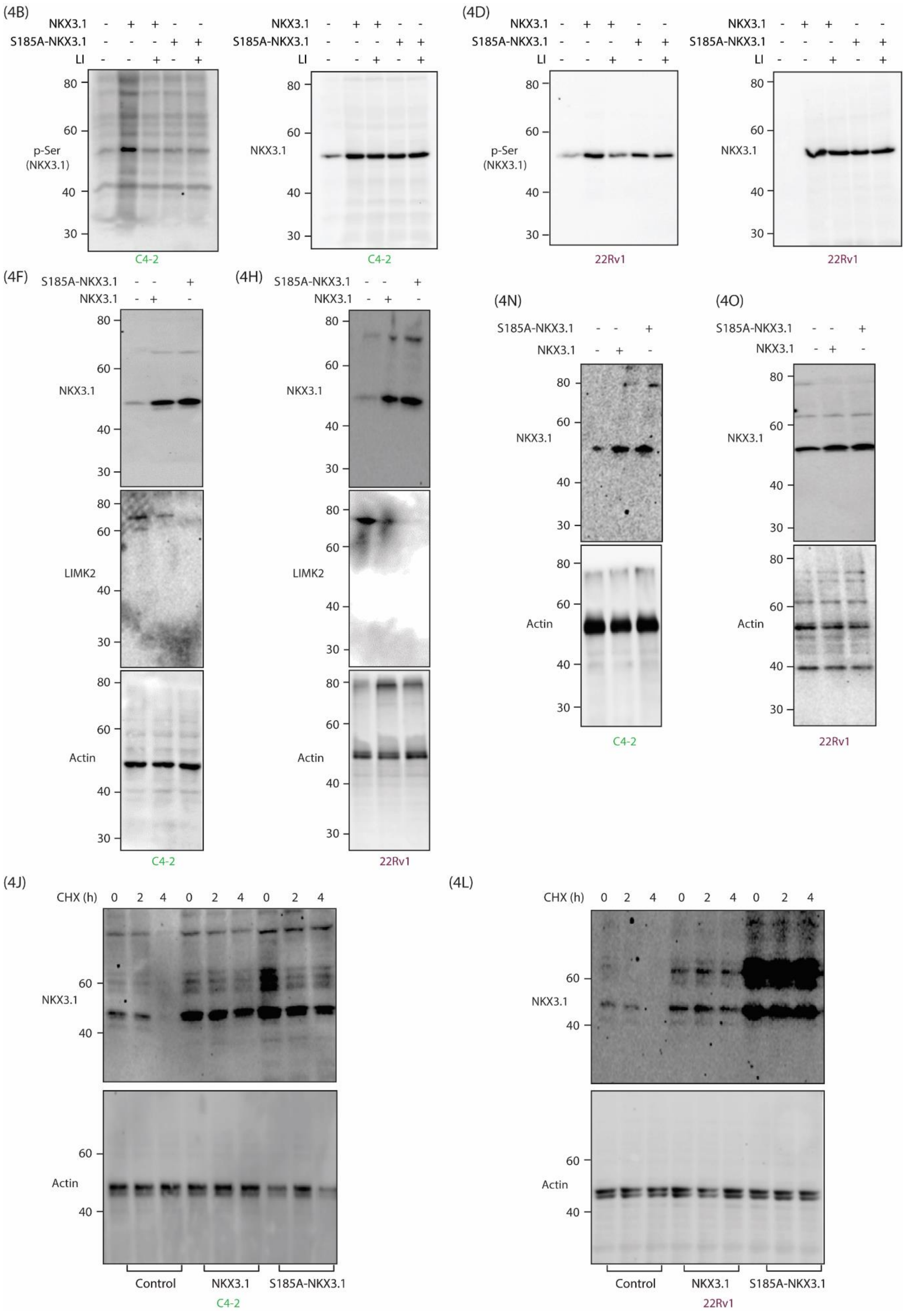
(4B)
NKX3.1
S185A-NKX3.1
LI
p-Ser
(NKX3.1)
NKX3.1
80
60
40
30
C4-2
C4-2
(4D)
NKX3.1
S185A-NKX3.1
LI
p-Ser
(NKX3.1)
NKX3.1
22Rv1
22Rv1
(4F)
S185A-NKX3.1
NKX3.1
NKX3.1
LIMK2
Actin
C4-2
(4H)
S185A-NKX3.1
NKX3.1
NKX3.1
LIMK2
Actin
22Rv1
(4N)
S185A-NKX3.1
NKX3.1
NKX3.1
Actin
C4-2
(4O)
S185A-NKX3.1
NKX3.1
NKX3.1
Actin
22Rv1
(4J)
CHX (h) 0 2 4 0 2 4 0 2 4
NKX3.1
Actin
Control
NKX3.1
S185A-NKX3.1
C4-2
(4L)
CHX (h) 0 2 4 0 2 4 0 2 4
NKX3.1
Actin
Control
NKX3.1
S185A-NKX3.1
22Rv1

(5A)

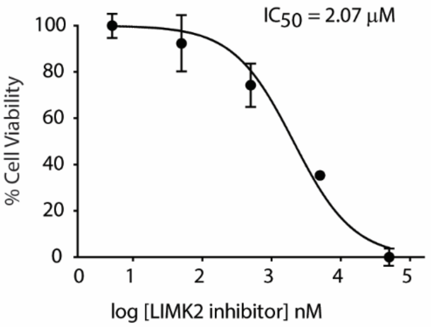

C4-2

(5B)

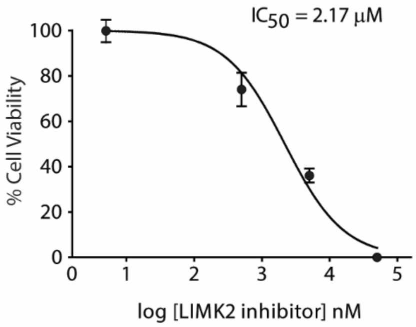

22Rv1

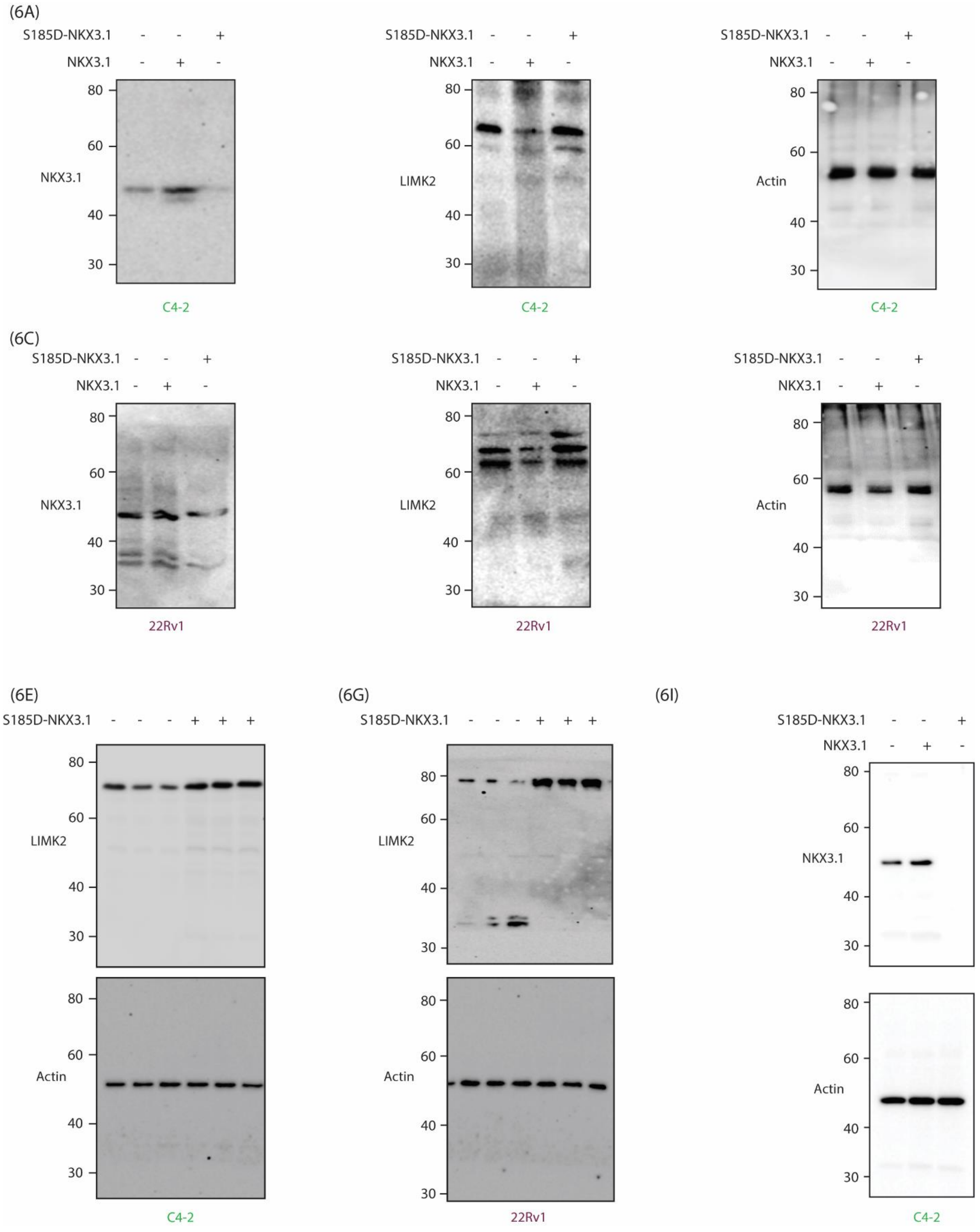
(6A)
S185D-NKX3.1 - - +
NKX3.1 - + -
80
60
40
30
NKX3.1
C4-2
S185D-NKX3.1 - - +
NKX3.1 - + -
80
60
40
30
LIMK2
C4-2
S185D-NKX3.1 - - +
NKX3.1 - + -
80
60
40
30
Actin
C4-2
(6C)
S185D-NKX3.1 - - +
NKX3.1 - + -
80
60
40
30
NKX3.1
22Rv1
S185D-NKX3.1 - - +
NKX3.1 - + -
80
60
40
30
LIMK2
22Rv1
S185D-NKX3.1 - - +
NKX3.1 - + -
80
60
40
30
Actin
22Rv1
(6E)
S185D-NKX3.1 - - - + + +
80
60
40
30
LIMK2
80
60
40
30
Actin
C4-2
(6G)
S185D-NKX3.1 - - - + + +
80
60
40
30
LIMK2
80
60
40
30
Actin
22Rv1
(6I)
S185D-NKX3.1 - - +
NKX3.1 - + -
80
60
40
30
NKX3.1
80
60
40
30
Actin
C4-2

(7A)

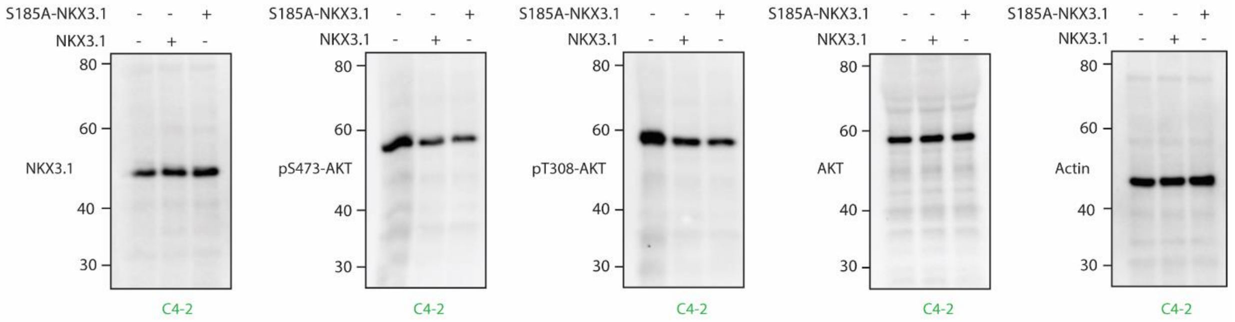
S185A-NKX3.1 - - +
NKX3.1 - + -
80
60
40
30
NKX3.1
pS473-AKT
pT308-AKT
AKT
Actin
C4-2

(7C)

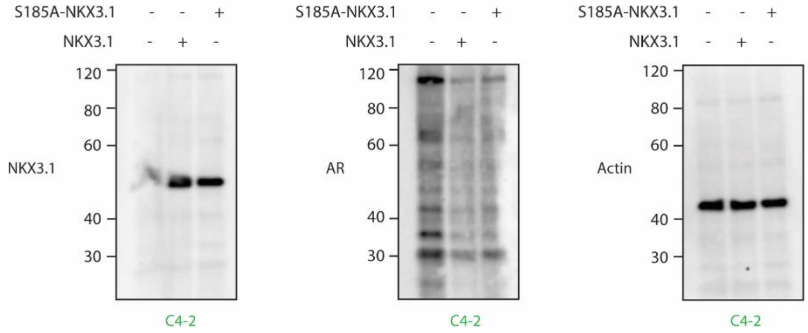
S185A-NKX3.1 - - +
NKX3.1 - + -
120
80
60
40
30
NKX3.1
AR
Actin
C4-2

(7E)

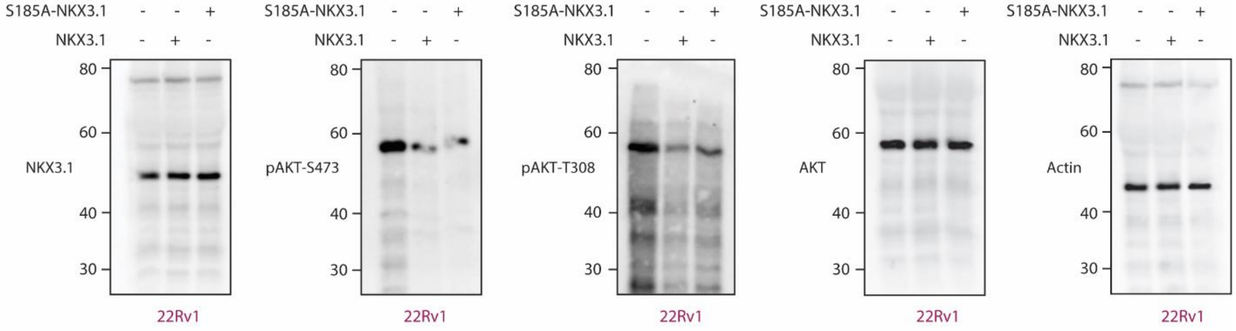
S185A-NKX3.1 - - +
NKX3.1 - + -
80
60
40
30
NKX3.1
pAKT-S473
pAKT-T308
AKT
Actin
22Rv1

(7G)

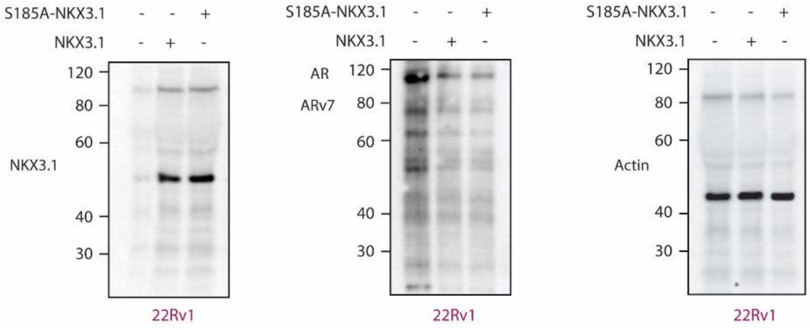
S185A-NKX3.1 - - +
NKX3.1 - + -
120
80
60
40
30
NKX3.1
AR
ARv7
Actin
22Rv1

(8A)

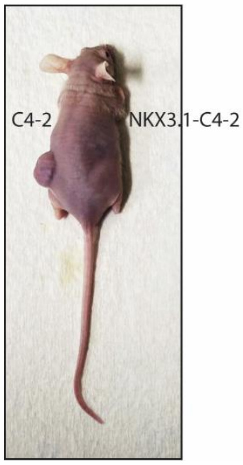
C4-2
NKX3.1-C4-2

(8B)

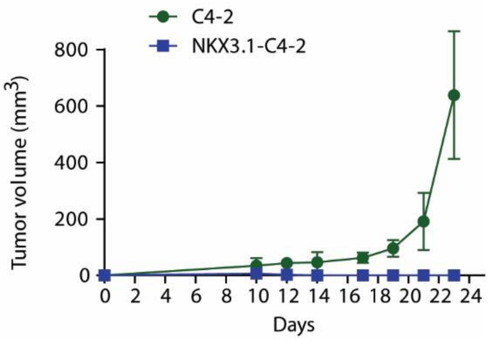
C4-2
NKX3.1-C4-2
Tumor volume (mm3)
800
600
400
200
0
0 2 4 6 8 10 12 14 16 18 20 22 24
Days
