# Supplementary material for: LIMK2-NKX3.1 Engagement Promotes Castration-Resistant Prostate Cancer
Source: Cancers (Basel). 2021 May 12;13(10):2324. doi: 10.3390/cancers13102324 (PMC8151535; doi:10.3390/cancers13102324)
Supplement: Supplementary file 1 [file cancers-13-02324-s001.zip › cancers-1173792-supplementary.pdf]

cancers

Article

LIMK2-NKX3.1 Engagement Promotes Castration-Resistant Prostate Cancer

Moloud A. Sooreshjani, Kumar Nikhil, Mohini Kamra, Dung N. Nguyen, Dinesh Kumar and Kavita Shah *

Department of Chemistry and Purdue University Center for Cancer Research, Purdue University, 560 Oval Drive, West Lafayette, IN 47907, USA; maflakis@purdue.edu (M.A.S.); niksbiotech@gmail.com (K.N.); mkamra@purdue.edu (M.K.); nguye445@purdue.edu (D.N.N.); kumar516@purdue.edu (D.K.)
* Correspondence: shah23@purdue.edu

Citation: Sooreshjani, M.A.; Nikhil, K.; Kamra, M.; Nguyen, D.N.; Kumar, D.; Shah, K. LIMK2-NKX3.1 Engagement Promotes Castration-Resistant Prostate Cancer. *Cancers* **2021**, *13*, 2324. https://doi.org/10.3390/cancers13102324

Academic Editor: Mohammad Saleem

Received: 22 March 2021
Accepted: 7 May 2021
Published: 12 May 2021

Publisher's Note: MDPI stays neutral with regard to jurisdictional claims in published maps and institutional affiliations.

Copyright: © 2021 by the authors. Licensee MDPI, Basel, Switzerland. This article is an open access article distributed under the terms and conditions of the Creative Commons Attribution (CC BY) license (http://creativecommons.org/licenses/by/4.0/).

Figure S1. (1D) The images represent the full-length western blot analysis of NKX3.1, Lamin A, and Actin from a fractionation experiment obtained from C4-2 cells treated by LIMK2 lentivirus. (1G) The images represent the full-length western blot analysis of NKX3.1, Lamin A, and Actin from a fractionation experiment obtained from 22Rv1 cells treated by LIMK2 lentivirus. (1N) The images represent the full-length western blot analysis of LIMK2, Lamin A, and Actin from a fractionation experiment obtained from C4-2 cells treated by NKX3.1 lentivirus. (1Q) The images represent the full-length western blot analysis of LIMK2, Lamin A, and Actin from a fractionation experiment obtained from 22Rv1 cells treated by NKX3.1 lentivirus.

Figure S2. (2A) The western blot images of Figure 2A. (2C) The western blot images of Figure 2C. (2E) The western blot images of Figure 2E. (2G) The western blot images of Figure 2G. (2H) The western blot images of Figure 2H. (2J) The western blot images of Figure 2J. (2O) The western blot images of Figure 2O. (2Q) Full-length western blot images of Figure 2Q. (2S) The western blot images of Figure 2S. (2T) The western blot images of Figure 2T.

Figure S3. (3A) The western blot images of Figure 3A. (3C) The western blot images of Figure 3C. (3E) the western blot images of Figure 3E. (3G) the western blot images of Figure 3G. (3M) The western blot images of Figure 3M. (3O) The western blot images of Figure 3O.

Figure S4. (4B) Full-length western blot images of Figure 4B. (4D) Full-length western blot images of Figure 4D. (4F) The western blot images of Figure 4F. (4H) The western blot images of Figure 4H. (4J) Full-length western blot images of Figure 4J. (4L) Full-length western blot images of Figure 4L. (4N) Full-length western blot images of Figure 4N. (4O). Full-length western blot images of Figure 4O.

Figure S5. (5A) Dose response curve of LIMK2 inhibitor in C4-2 cells. Different doses of LIMK2 inhibitor were used (10 nM, 100 nM, 1 µM, 10 µM and 100 µM). DMSO-treated cells were used as control. Cell viability was analyzed after 48 h. (B) Dose response curve of LIMK2 inhibitor in 22Rv1 cells.

Figure S6. (6A) Full-length western blot images of Figure 6A. (C) Full-length western blot images of Figure 6C. (6E) Full-length western blot images of Figure 6E. (6G) Full-length western blot images of Figure 6G. (6I) Full-length western blot images of Figure 6I.

Figure S7. (7A) Full-length western blot images of Figure 7A. (7C) Full-length western blot images of Figure 7C. (7E) Full-length western blot images of Figure 7E. (7G) Full-length western blot images of Figure 7G.

Figure S8. (A) Representative image of nude mouse bearing xenografts, 23 days after subcutaneous injection with C4-2 (left) and NKX3.1-C4-2 (right) cells. (B) Tumor volumes were monitored over 3 weeks post-inoculation and plotted as mean ± SEM.

Table S1. Details of antibodies details used in this study.

No.		Catalog No.	Company	RRID
1	NKX3.1	SC-393190	Santa Cruz Biotech	
2	Actin	SC-8432	Santa Cruz Biotech	RRID:AB_626630
3	LIMK2	SC-365414	Santa Cruz Biotech	RRID: AB_10847817
4	HA	12CA5	Thermo Fisher	RRID:AB_1958069
5	6x-His	AB-1711	Columbia Bioscience	Lot# PUR01016008
6	AR	SC-816	Santa Cruz Biotech	RRID:AB_ 1563391
7	ARv7	M00542-3	BosterBio	
8	Lamin-A	SC-20680	Santa Cruz Biotech	RRID:AB_648148
9	NKX3.1	83700S	Cell Signaling	RRID: AB_2800027
10	AKT	9272	Cell Signaling	RRID:AB_329828
11	pS473-AKT	SC-293125	Santa Cruz Biotech	RRID: AB_2847909
12	pT308-AKT	9275S	Cell Signaling	RRID:AB_329828
13	Phospho-Ser	sc-81514	Santa Cruz Biotech	RRID:AB_1128624

Table S2. Sequences of NKX3.1 shRNA.

Names	Primer Sequences
NKX3.1 shRNA (forward)	CCG GAA GTT CAG CCA TCA GAA GTA CTC GAG TAC TTC TGA TGG CTG AAC TTC TTT TTG
NKX3.1 shRNA (reverse)	AAT TCA AAA AAA GTT CAG CCA TCA GAA GTA CTC GAG TAC TTC TGA TGG CTG AAC TTC

Table S3. Details of qPCR primers.

Names	Primer Sequences
AURKA-F	5'- CCACCTTCGGCATCCTAATA -3'
AURKA-R	5'- TCCAAGTGGTGCATATTCCA -3'
YBX1-F	5'- GGAGTTTGATGTTGTTGAAGGA -3'
YBX1-R	5'- AACTGGAACACCACCAGGAC -3'
AR-F	5'- AAGACGCTTCTACCAGCTCACCAA -3'
AR-R	5'- TCCCAGAAAGGATCTTGGGCACTT -3'
ARv7-F	5'- CAGGGATGACTCTGGGAGAA -3'
ARv7-R	5'- GCCCTCTAGAGCCCTCATTT -3'
RAMP1-F	5'- CGCACACGATTGGCTGTTT -3'
RAMP1-R	5'- TGGACAGCGATGAAGAATCTGT -3'
β-Actin-F	5'- CATGTACGTTGCTATCCAGGC -3'
β-Actin-R	5'- CTCCTTAATGTCACGCACGAT -3'
